# Polo like kinase 1 expression in cervical cancer tissues generated from multiple detection methods

**DOI:** 10.7717/peerj.10458

**Published:** 2020-12-08

**Authors:** Li Gao, Yu-Yan Pang, Xian-Yu Guo, Jing-Jing Zeng, Zhong-Qing Tang, Dan-Dan Xiong, Xia Yang, Ying Li, Fu-Chao Ma, Lin-Jiang Pan, Zhen-Bo Feng, Gang Chen

**Affiliations:** 1 Department of Pathology, The First Affiliated Hospital of Guangxi Medical University, Nanning, Guangxi Zhuang Autonomous Region, China; 2 Department of Radiotherapy, The First Affiliated Hospital of Guangxi Medical University, Nanning, Guangxi Zhuang Autonomous Region, China; 3 Department of Pathology, Wuzhou Gongren Hospital / The Seventh Affiliated Hospital of Guangxi Medical University, Wuzhou, Guangxi Zhuang Autonomous Region, China; 4 Department of Pathology, Qinzhou First People’s Hospital, Qinzhou, Guangxi Zhuang Autonomous Region, China; 5 Department of Medical Oncology, The First Affiliated Hospital of Guangxi Medical University, Nanning, Guangxi Zhuang Autonomous Region, China

**Keywords:** Cervical cancer, Tissue microarray, RNA-seq, Prognosis, Clinico-pathological significance, CESC, Cervical adenocarcinoma, Genetic alteration, Pathway analysis

## Abstract

**Background:**

Existing studies of PLK1 in cervical cancer had several flaws. The methods adopted by those studies of detecting PLK1 expression in cervical cancer were single and there lacks comprehensive evaluation of the clinico-pathological significance of PLK1 in cervical cancer.

**Methods:**

A total of 303 cervical tissue samples were collected for in-house tissue microarrays. Immunohistochemistry was performed for evaluating PLK1 expression between cervical cancer (including cervical squamous cell carcinoma (CESC) and cervical adenocarcinoma) and non-cancer samples. The Expression Atlas database was searched for querying PLK1 expression in different cervical cancer cell lines and different tissues in the context of pan-cancer. Standard mean difference (SMD) was calculated and the summarized receiver’s operating characteristics (SROC) curves were plotted for integrated tissue microarrays, exterior high-throughput microarrays and RNA sequencing data as further verification. The effect of PLK1 expression on the overall survival, disease-free survival and event-free survival of cervical cancer patients was analyzed through Kaplan Meier survival curves for cervical cancer patients from RNA-seq and GSE44001 datasets. The gene mutation and alteration status of PLK1 in cervical cancer was inspected in COSMIC and cBioPortal databases. Functional enrichment analysis was performed for genes correlated with PLK1 from aggregated RNA-seq and microarrays.

**Results:**

A total of 963 cervical cancer samples and 178 non-cancer samples were collected from in-house tissue microarrays and exterior microarrays and RNA-seq datasets. The combined expression analysis supported overexpression of PLK1 in CESC, cervical adenocarcinoma and all types of cervical cancer (SMD = 1.59, 95%CI [0.56–2.63]; SMD = 2.99, 95%CI [0.75–5.24]; SMD = 1.57, 95% CI [0.85–2.29]) and the significant power of PLK1 expression in distinguishing CESC or all types of cervical cancer samples from non-cancer samples (AUC = 0.94, AUC = 0.92). Kaplan-Meier survival curves showed that the event-free survival rate of cervical cancer patients with higher expression of PLK1 was shorter than that of patients with lower PLK1 (HR = 2.020, *P* = 0.0197). Genetic alteration of PLK1 including missense mutation and mRNA low occurred in 6% of cervical cancer samples profiled in mRNA expression. Genes positively or negatively correlated with PLK1 were mainly assembled in pathways such as DNA replication, cell cycle, mismatch repair, Ras signaling pathway, melanoma, EGFR tyrosine kinase inhibitor resistance and homologous recombination (*P* < 0.05).

**Conclusions:**

Here, we provided sufficient evidence of PLK1 overexpression in cervical cancer. The overexpression of PLK1 in cervical cancer and the contributory effect of it on clinical progression indicated the hopeful prospect of PLK1 as a biomarker for cervical cancer.

## Introduction

Cervical cancer is one of the most common gynecologic tumors in women (Siegel, Miller & Jemal, 2018). In developing countries, due to the lack of health care consciousness and the relative shortage of medical resources, cervical cancer is still seriously endangering women’s health and the mortality rate of cervical cancer increased with age (Jiang, Tang & Chen, 2018). The new incidence of cervical cancer in China accounts for approximately one-fourth of the total number of new cases of cervical cancer globally and an estimated number of 30,500 deaths of cervical cancer occurred annually in China (Wei et al., 2019). The occurrence and development of cancer could be attributed by the activation of oncogene and inactivation of tumor suppressor genes under the action of many factors. Thus, focusing on oncogenes or tumor suppressor genes with clinical significance in cervical cancer might facilitate understanding the molecular mechanism of cervical cancer and discovering effective biomarkers.

PLK1 (polo like kinase 1) is a highly conserved serine/threonine protein kinase widely existing in eukaryotic cells with diverse functions of regulating cell cycle, inhibiting tumor cell apoptosis and promoting tumor formation. PLK1 was reported to promote the abnormalities of early cervical columnar epithelial cells (Oh et al., 2014; Yang et al., 2017). The overexpression of PLK1 was found in various types of cancers and was associated with poor prognosis of cancer patients (Weng Ng et al., 2016; Lin et al., 2017; Li et al., 2017; Dang et al., 2018). Although several researches involved PLK1 in cervical cancer (Yang et al., 2017; Li et al., 2011; Zhang et al., 2009; Gao et al., 2006; Zuco et al., 2015; Chhavi et al., 2010), PLK1 expression in cervical cancer was detected with single methods and small samples by those studies. None of these studies compared PLK1 expression in both cervical squamous cell carcinoma versus non-cancer cervix tissues and cervical adenocarcinoma versus non-cancer cervix tissues. These studies also lacked comprehensive evaluation of the clinico-pathological significance of PLK1 in cervical cancer and the molecular mechanism underlying PLK1 in cervical cancer has not been elucidated. In this study, the differential expression of PLK1 between cervical cancer and non-cancer tissues as well as the clinico-pathological significance of PLK1 in cervical cancer was evaluated by combined methods of tissue microarray, immunohistochemistry (IHC), Expression Atlas and further verified by standard mean difference (SMD) calculated for high-throughput microarrays and RNA sequencing data. The molecular mechanism beneath PLK1 in cervical cancer was further explored via analyzing the genetic alteration profile of PLK1 in cervical cancer and functional enrichment of PLK1-related genes in cervical cancer.

## Materials & Methods

### Tissue microarray

#### Research object

A total of 303 cervical tissue samples were collected by Hangzhou panspectrum Biotechnology Co., Ltd. (hereinafter referred to as Panspectral Biology), including 290 cases of cervical cancer tissue and 13 cases of noncancer cervical tissue (including cervicitis, polyps and condyloma acuminatum), which were collected from July 2018 to August 2018. According to International Federation of Gynecology and Obstetrics (FIGO), there were 23 cases in stage I, 126 cases in stage II and 115 cases in stage III. According to the TNM staging issued by American Joint Committee on Cancer (AJCC) and Union International Center of Cancer (UICC), there were 257 cases in T1, 29 cases in T2, one case in T3, two cases in T4, 214 cases in N0 and 75 cases in N1-2. No distant metastasis was found in all included cases. The study was approved by the ethics committee of the First Affiliated Hospital of Guangxi Medical University (2020(KY-E-095)) and all patients signed informed consent.

#### Inclusion and exclusion criteria of research objects

Inclusion criteria: 1. All cervical cancer samples were collected from surgical resection specimens by Panspectrum Biotechnology Co., Ltd. and the diagnosis was confirmed by pathological examination. 2. The cervical cancer patients did not receive radiotherapy, chemotherapy, immunotargeting therapy or other related treatment. 3. The pathological examination results and medical records of the included subjects were complete. Exclusion criteria: 1. The cervical cancer patients developed distant metastasis. 2. The cervical cancer patients had concomitant other tumors. 3. The clinical data of patients were incomplete for statistical analysis. 4. No pathological diagnosis report was obtained from the patients.

#### IHC

Sections from paraffin-embedded (FFPE) specimens of all included samples were deparaffinized and rehydrated, followed by repeated washing with ionic water. Then the milk liquid was added for blocking antibody and the blocking time was 5 min. The sections were added with antibody for PLK1 (rabbit polyclonal antibody, dilution 1:500, Abcam’s rabmab Technology) and incubated at 37 °C for 2 h. Sections were washed three times with phosphate buffer and treated by fluorescent labeled secondary antibody (purchased from Abcam company) followed by incubation at 37 °C for 30 min. The sections were washed with phosphate buffer solution, stained by chromogenic agent and cover-slipped for microscopic observation.

The sections were evaluated by two experienced pathologists independently. If two pathologists reported inconsistent scores, a third pathologist should be asked to review the results. The staining intensity scores ranged from 0–3 with 0 for no staining, 1 for weak staining, 2 for moderate staining and 3 for strong staining when brown signals appeared in the cell cytoplasm or nucleus. Percentage of positively stained cells were judged as 0 for ≦ 5%, 1 for 6%–25%, 2 for 26%–50%, 3 for 51%–75% and 4 for > 75%. The final IHC scores were the multiplying of the two parts. The IHC scores of 0–3 were considered as negative staining (-), scores of 4-5 were considered as weak staining (+), scores of 6 - 9 were considered as moderate staining (++) and scores of > 9 were considered as strong staining (+ + +). Low expression corresponded to negative or weak staining while high expression corresponded to moderate or strong staining.

### Validation of PLK1 expression from the Expression Atlas database

Expression Atlas is an added value database with plentiful information of gene and protein expression across various species in different tissues, diseases and cell types (https://www.ebi.ac.uk/gxa/home) (Papatheodorou et al., 2018). Data sets from different sources are curated and re-analyzed using standardized pipelines and it is available for users to query, download or visualize processed data (Papatheodorou et al., 2018). In the present study, we searched PLK1 expression in 17 different cervical cancer cell lines and we also compared PLK1 expression in different tissues in the context of pan-cancer.

### Verification from external microarrays and RNA-seq datasets

Fragments per kilobase per million (FPKM) gene expression matrix and supporting clinical information of cervical cancer patients in The Cancer Genome Atlas (TCGA) were downloaded from Genomic Data Commons (GDC) data portal (https://portal.gdc.cancer.gov/repository). We also downloaded the transcripts perkilobase million (TPM) gene expression matrix of normal cervical tissues from Genotype-Tissue Expression (GTEx) database to supplement the normal cervical samples of TCGA database. Datasets from TCGA and GTEx (253 cervical squamous cell carcinoma (CESC) samples, 53 cervical adenocarcinoma samples and 14 non cancer samples) were combined and the gene expression matrix was standardized as the form of log2 (TPM + 0.001). Additionally, microarrays from gene expression omnibus (GEO) (https://www.ncbi.nlm.nih.gov/gds/?term=) or ArrayExpress (https://www.ebi.ac.uk/arrayexpress/) databases containing expression data of PLK1 in no less than three human cervical cancer and non cancer cervical samples published before May 19, 2020 were also included for expression analysis.

Expression data of PLK1 from microarrays were extracted and processed as described before (Gao et al., 2018; Yang et al., 2018). The standardized mean difference (SMD) and 95% confidence interval (CI) of PLK1 expression between cervical cancer and non-cancer samples were calculated for all included microarrays and RNA-seq datasets, with the methods of random-effect model of meta package in R software v.3.6.1. The summarized receiver’s operating characteristics (SROC) curves were drawn by CopulaREMADA, INLA and meta4diag packages in R software v.3.6.1 for appraising the ability of PLK1 to differentiate cervical cancer from non-cancer samples (Ren et al., 2018).

### Integration of tissue microarray, external microarrays and RNA-seq datasets for comprehensive PLK1 expression analysis

The IHC scores of PLK1 in cervical cancer and non-cancer tissues from the experiments of tissue microarray were merged with expression matrix from external microarrays and RNA-seq datasets for comprehensive evaluation of PLK1 expression in CESC versus non-cancer tissues, cervical adenocarcinoma versus non-cancer tissues or all types of cervical cancer versus non-cancer tissues. Expression data of PLK1 including the number of cancer and non-cancer samples, average IHC scores of PLK1 in cancer and non-cancer samples and the standard deviation of IHC scores of PLK1 in cancer and non-cancer samples as well as the diagnostic data of tissue microarray were added to the matrix of external microarray and RNA-seq datasets. SMD and SROC curves were calculated for merged expression matrix of PLK1 in CESC versus non-cancer tissues, cervical adenocarcinoma versus non-cancer tissues and all types of cervical cancer versus non-cancer tissues. The methods of plotting SMD forest plot and SROC curves were stated above.

### Survival analysis

RNA-seq or microarrays from TCGA, GEO and ArrayExpress databases were included for survival analysis if survival information of cervical cancer patients and PLK1 expression data in cervical cancer tissues were provided. The effect of PLK1 expression on the overall survival, disease-free survival, or event-free survival of cervical cancer patients from included datasets was analyzed through Kaplan Meier survival curves with log-rank *P* value and hazard ratio (HR) calculated by GraphpadPrism v.8.0.1. Cutoff value was set as the median of expression value of PLK1 and *P* < 0.05 indicated statistical significance.

### Mutation and alteration status of PLK1 in cervical cancer

Catalogue of Somatic Mutations in Cancer (COSMIC) and cBioPortal databases were employed for querying mutation types of PLK1 and genetic alteration profile of PLK1 in 190 cervical cancer samples with mutation data from TCGA database. We searched the COSMIC database with the keyword: “PLK1” and downloaded the mutation distribution part. Similarly, we selected TCGA Cervical Squamous Cell Carcinoma and Endocervical Adenocarcinoma Source data from GDAC Firehose and input the keyword: “PLK1” in the search box to investigate the mutation status and mRNA expression *z*-scores relative to all samples (log RNA Seq V2 RSEM) of PLK1 in cervical cancer samples.

### Functional enrichment analysis for PLK1-correlated genes in cervical cancer

Firstly, we collected RNA-seq dataset from TCGA database and microarrays with gene expression matrix of cervical cancer from GEO or ArrayExpress databases. Gene expression matrix of CESC versus non-cancer cervix tissues and cervical adenocarcinoma versus non-cancer tissues from all included datasets were prepared separately and normalized using limma package in R software v.3.6.1. Differential expression analysis was conducted for each expression matrix with limma package in R software v.3.6.1 and genes concurrently displayed upregulation or downregulation (log2FC >1&adjusted *P* <  0.05; log2FC <-1&adjusted *P* > 0.05) in at least two datasets of cervical adenocarcinoma, genes concurrently displayed upregulation (log2FC >1&adjusted *P* <0.05) in at least three datasets of CESC and genes concurrently displayed downregulation (log2FC <-1&adjusted *P* < 0.05) in at least two datasets of cervical squamous cell carcinoma were defined as differentially expressed genes (DEGs) of cervical cancer. Expression correlation analysis was carried out using the Pearson correlation method of psych package in R software v.3.6.1 for each qualified dataset. The intersection of the above defined upregulated DEGs and genes concurrently showed positive correlation with PLK1 (*r* > 0, adjusted *P* < 0.05) in at least one dataset of cervical adenocarcinoma were designated as genes positively correlated with PLK1 in cervical adenocarcinoma; the intersection of the above defined downregulated DEGs and genes concurrently showed negative correlation with PLK1 (*r* < 0, adjusted *P* < 0.05) in at least one dataset of CESC were designated as genes negatively correlated with PLK1 in CESC. The intersection of the above defined upregulated DEGs and genes concurrently showed positive correlation with PLK1 (*r* > 0, adjusted *P* <0.05) in at least three dataset of CESC were designated as genes positively correlated with PLK1 in CESC; the intersection of the above defined downregulated DEGs and genes concurrently showed negative correlation with PLK1 (*r* < 0, adjusted *P* < 0.05) in at least two dataset of CESC were designated as genes negatively correlated with PLK1 in CESC. Gene ontology (GO) and Kyoto Encyclopedia of Genes and Genomes (KEGG) pathway analysis was performed for genes positively or negatively correlated with PLK1 via ClusterProfiler package in R software v.3.6.1. Significant GO and KEGG terms should meet the criteria of adjusted *P* < 0.05.

### Statistical analysis

SPSS 16.0 was employed for the statistical analysis of in-house tissue microarray data. IHC data of PLK1 expression in cervical cancer and non-cancer tissues were presented as binary data. Chi-square test was used for comparing PLK1 protein expression in different clinical parameter groups. *P* < 0.05 was considered as statistically significant.

## Results

### Clinical significance of PLK1 in cervical cancer tissues from tissue microarrays

**Figure 1:**
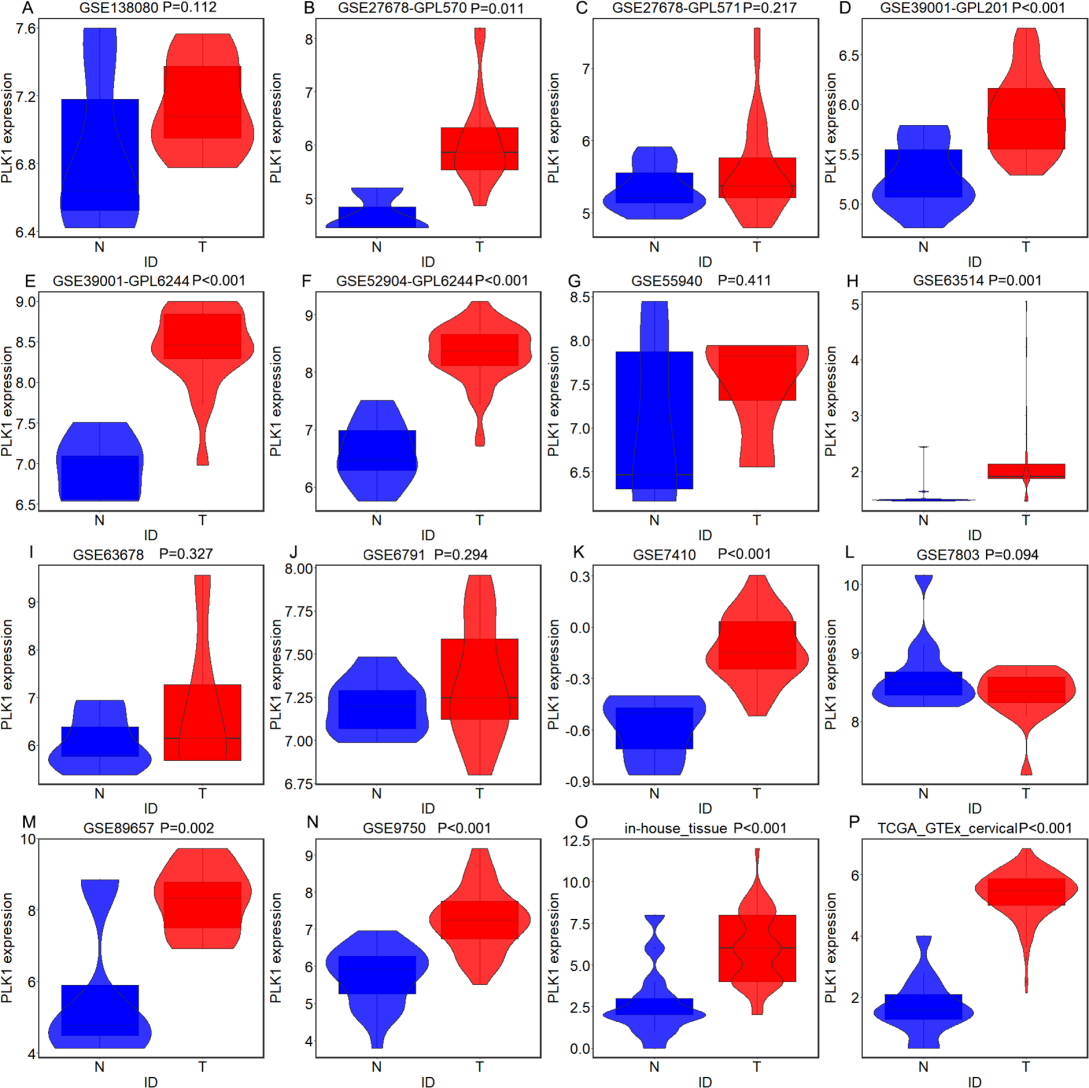
PLK1 expression in all types of cervical cancer and non-cancer samples from in-house tissue microarrays, external microarrays and RNA-seq dataset. (A) Violin plots for GSE138080; (B) Violin plots for GSE27678 (GPL570); (C) Violin plots for GSE27678 (GPL571); (D) Violin plots for GSE39001 (GPL201); (E) Violin plots for GSE39001 (GPL6244); (F) Violin plots for GSE55940; (G) Violin plots for GSE63514; (H) Violin plots for GSE63678; (I) Violin plots for GSE6791; (J) Violin plots for GSE7410; (K) Violin plots for GSE7803; (L) Violin plots for GSE89657; (M) Violin plots for GSE9750; (N) Violin plots for GSE52904 (GPL6244); (O) Violin plots for TCGA-GTEx datasets; (P) Violin plots for in-house tissue microarrays. The expression shown in blue is for individuals without cancer, and the expression shown in red is for individuals with cancer.

**Figure 2:**
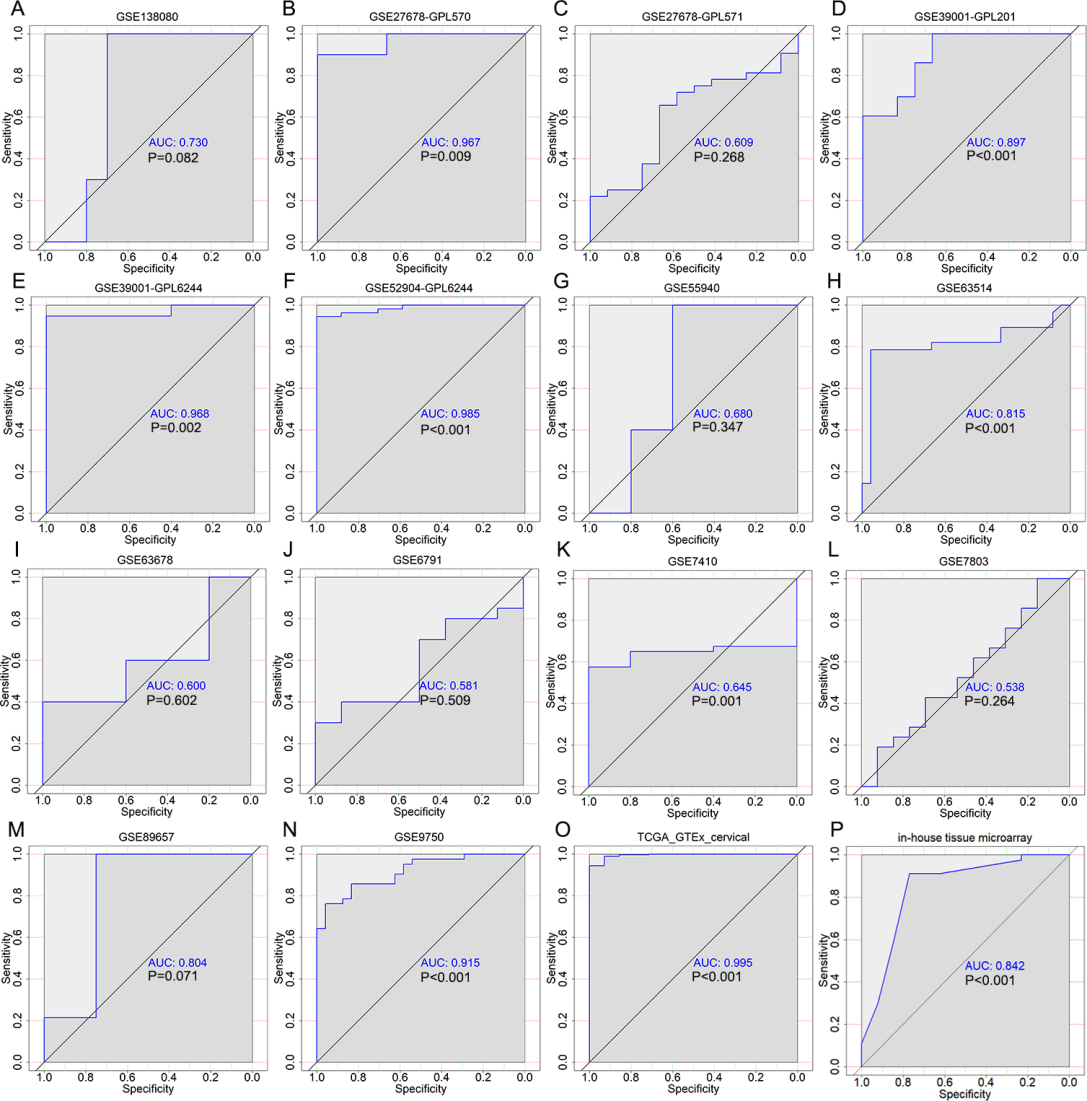
The discriminatory ability of PLK1 expression in distinguishing cervical cancer from non-cancer tissues in each microarray and RNA-seq dataset. (A) ROC curves for GSE6791; (B) ROC curves for GSE7410; (C) ROC curves for GSE7803; (D) ROC curves for GSE9750; (E) ROC curves for GSE27678 (GPL570); (F) ROC curves for GSE27678 (GPL571); (G) ROC curves for GSE39001 (GPL201); (H) ROC curves for GSE39001 (GPL6244); (I) ROC curves for GSE55940; (J) ROC curves for GSE63514; (K) ROC curves for GSE63678; (L) ROC curves for GSE89657; (M) ROC curves for GSE138080; (N) ROC curves forGSE52904 (GPL6244); (O) ROC curves for TCGA-GTEx datasets; (P) ROC curves for in-house tissue microarray.

**Figure 3:**
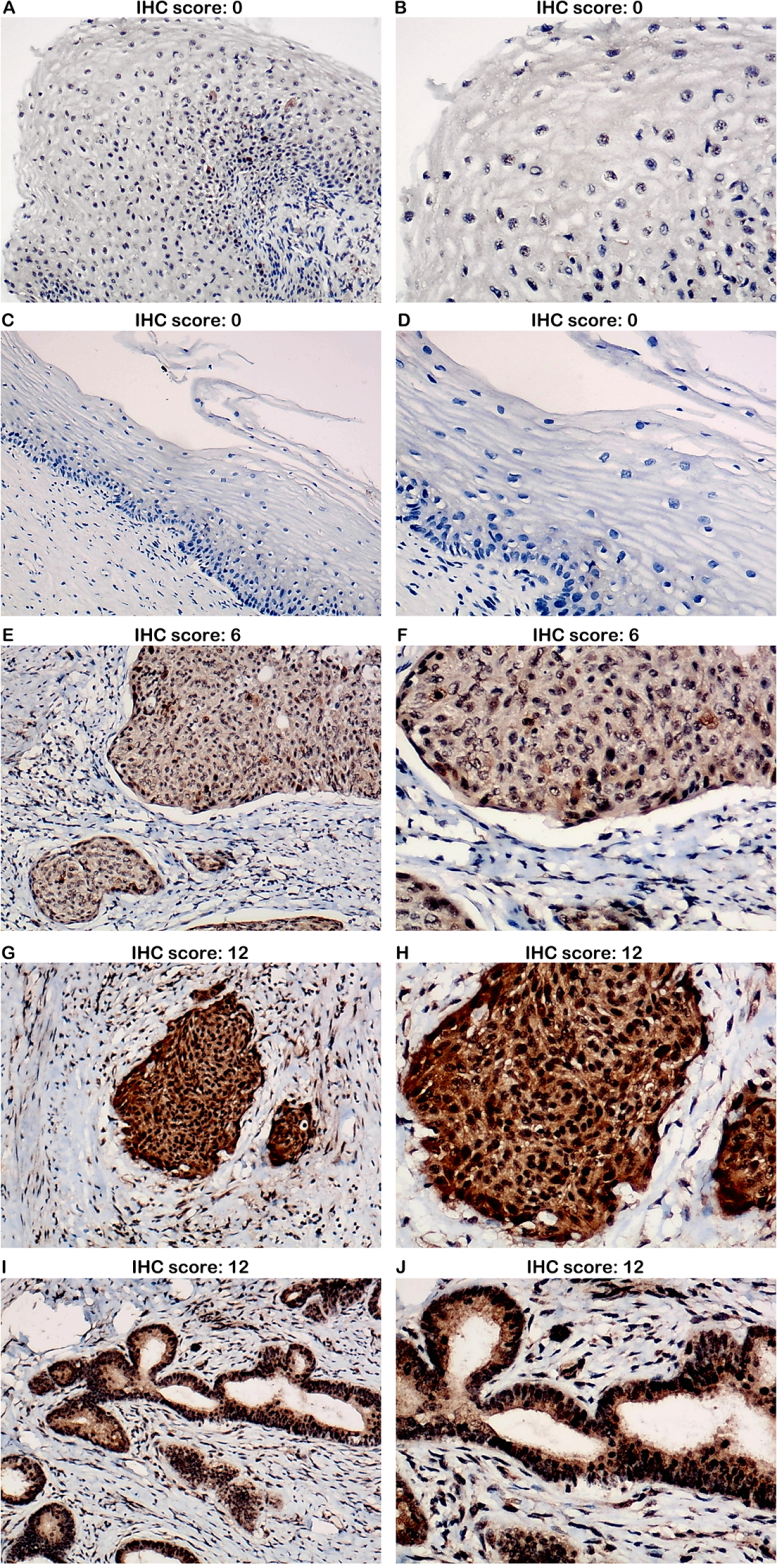
IHC staining of PLK1 in cervical cancer and non-cancer tissues from tissue microarrays. (A) Negative staining of PLK1 in non-cancer tissues (100x); (B) Negative staining of PLK1 in non-cancer tissues (200x); (C) Negative staining of PLK1 in non-cancer tissues (100x); (D) Negative staining of PLK1 in non-cancer tissues (200x); (E) Moderate staining of PLK1 in cervical squamous cell carcinoma tissues (100x); (F) Moderate staining of PLK1 in cervical squamous cell carcinoma tissues (200x); (G) Strong staining of PLK1 in cervical squamous cell carcinoma tissues (100x); (H) Strong staining of PLK1 in cervical squamous cell carcinoma tissues (200x); (I) Strong staining of PLK1 in cervical adenocarcinoma tissues (100x); (J) Strong staining of PLK1 in cervical adenocarcinoma tissues (200x).

According to the expression data of PLK1 in 290 cervical cancer tissues and 13 non-cancer tissues, PLK1 presented significantly higher protein expression in cervical cancer tissues than in non-cancer tissues (X^2^ = 11.108, *P* = 0.001) (Fig. 1O) (Table 1). Overexpression of PLK1 could well distinguish cervical cancer from non-cancer tissues (AUC = 0.842, *P* < 0.001) (Fig. 2P). Among the collected 290 cervical cancer specimens, there were 19 cases (6.6%) with negative staining, 92 cases (31.7%) with weak staining, 172 cases (59.3%) with moderate staining and 7 cases (2.4%) with strong staining. Moderate and even strong immunostaining of PLK1 could be observed in 61.7% of cervical cancer samples while 84.6% of non-cancer cervix samples presented negative or weak immunostaining of PLK1 (Fig. 3). A total of 226 CESC samples and 63 cervical adenocarcinoma samples were included in 290 cervical cancer samples. Comparison of PLK1 expression between CESC and cervical adenocarcinoma tissues revealed no significant difference (X^2^ = 2.889, *P* = 0.089, Table 1). PLK1 exhibited concurrent upregulation in CESC or cervical adenocarcinoma samples compared with non-cancer tissues (Figs. S1 and S2) (Tables 2 and 3), which was a significant feature in the discrimination of cancer from non-cancer tissues (Figs. S3 and S4). Remarkably higher expression of PLK1 was observed in all types of cervical cancer patients with lymph node metastasis or CESC patients with lymph node metastasis than in those without lymph node metastasis (X^2^ = 29.859, *P* < 0.001; X^2^ = 29.324, *P* < 0.001) (Tables 1 and 2). No significant relationship between PLK1 expression and other clinical variables of cervical cancer was found.

**Table 1:** The clinic-pathological significance of PLK1 in cervical cancer.

Clinico-pathological variables	High PLK1 expression		Low PLK1 expression		*χ*^2^	*P*
	Number of cases %		Number of cases %			
Tissue type					11.108	0.001
Cervical cancer	179	61.7	111	38.3		
Non-cancer	2	15.4	11	84.6		
Subtype						
CESC	145	64.2%	81	35.8%	2.889^*^	0.089
Cervical adenocarcinoma	33	52.4%	30	47.6%		
Malignant mullerian mixed tumor	1	100%	0	0%		
Age					0.656	0.418
≤44	88	59.5	60	40.5		
>44	91	64.1	51	35.9		
FIGO stage					1.862	0.172
I+II	99	66.4	50	33.6		
III	67	58.3	48	41.7		
T stage					1.89	0.169
T1+T2	175	61.2	111	38.8		
T3+T4	3	100.0	0	0.0		
N stage					29.859	<0.001
N0	112	52.3	102	47.7		
N1-2	66	88.0	9	12.0		

**Notes.**

FIGO: Federation of Gynecology and Obstetrics.

Chi-square test was used for comparing PLK1 protein expression in different clinical parameter groups.

* Comparison of PLK1 protein expression in different subtypes of cervical cancer was made between CESC and cervical adenocarcinoma.

**Table 2:** The clinic-pathological significance of PLK1 in cervical squamous cell carcinoma (CESC).

Clinico-pathological variables	High PLK1 expression		Low PLK1 expression		*χ*^2^	*P*
	Number of cases %		Number of cases %			
Tissue type					12.352	<0.001
CESC	145	64.2	81	35.8		
Non-cancer	2	15.4	11	84.6		
Age					2.903	0.088
≤44	67	58.8	47	41.2		
>44	78	69.6	34	30.4		
Grade					3.581	0.167
I	12	80.0	3	20.0		
II	69	67.6	33	32.4		
III	64	58.7	45	41.3		
T stage					0.486	0.486
T1+T2	142	63.7	81	36.3		
T3+T4	3	100.0	0	0.0		
N stage					29.324	<0.001
N0	90	53.9	77	46.1		
N1-N2	55	93.2	4	6.8		

**Notes.**

FIGO, Federation of Gynecology and Obstetrics.

Chi-square test was used for comparing PLK1 protein expression in different clinical parameter groups.

**Table 3:** The clinic-pathological significance of PLK1 in cervical adenocarcinoma.

Clinico-pathological variables	High PLK1 expression		Low PLK1 expression		*χ*^2^	*P*
	Number of cases %		Number of cases %			
Tissue type					5.937	0.015
Cervical adenocarcinoma	33	52.4	30	47.6		
Non-cancer	2	15.4	11	84.6		
Age					2.607	0.106
≤44	21	61.8	13	38.2		
>44	12	41.4	17	58.6		
Grade					0.407	0.903
I	4	50.0	4	50.0		
II	14	58.3	10	41.7		
III	3	50.0	3	50.0		
T stage					NA	NA
T1+T2	33	52.4	30	47.6		
T3+T4	0	0.0	0	0.0		
N stage					2.304	0.129
N0	22	46.8	25	53.2		
N1-N2	11	68.8	5	31.3		

**Notes.**

FIGO, Federation of Gynecology and Obstetrics.

Chi-square test was used for comparing PLK1 protein expression in different clinical parameter groups.

### Validation of PLK1 expression from Expression Atlas database

**Figure 4:**
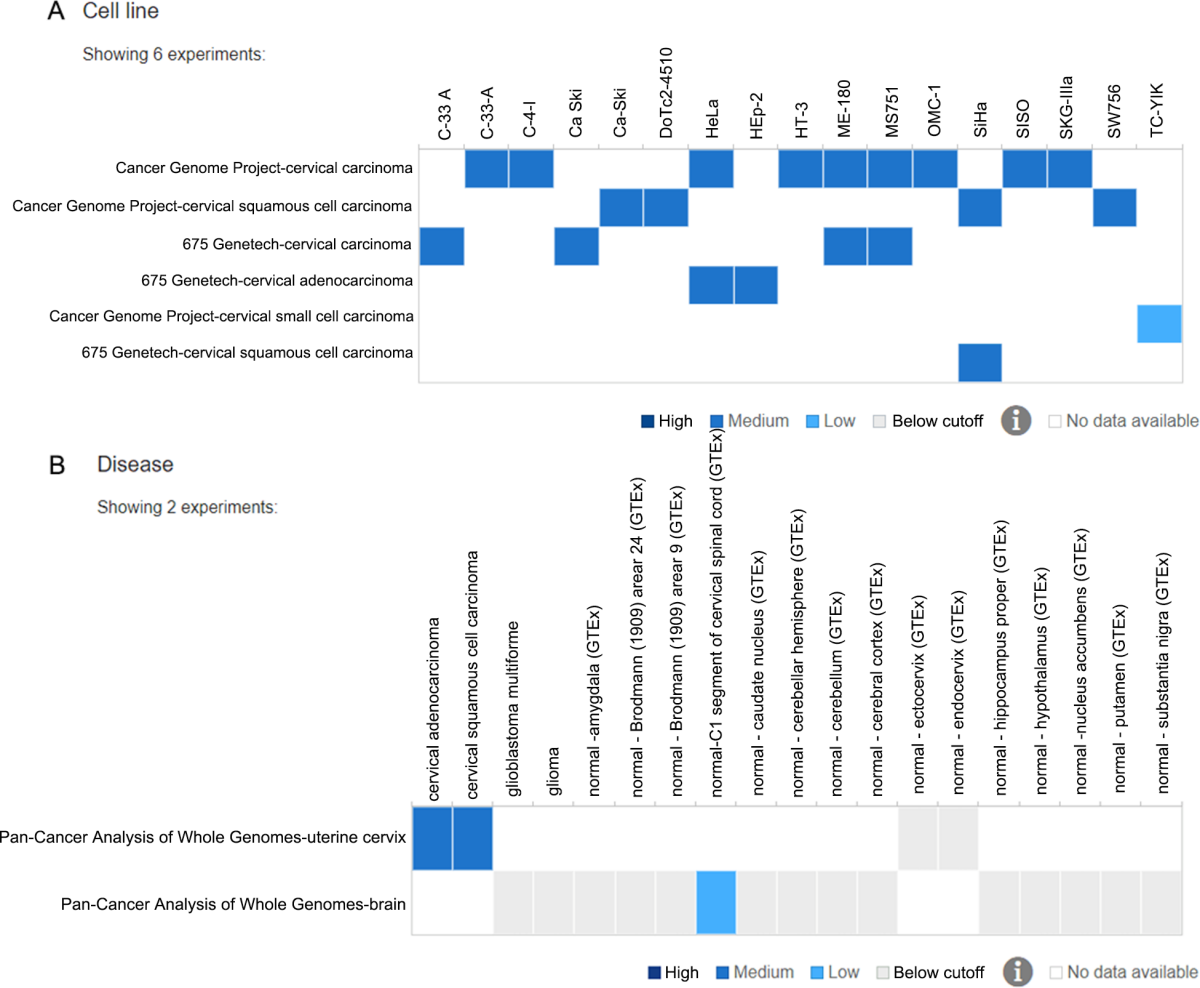
PLK1 expression in different cervical samples from the Expression Atlas. (A) PLK1 expression in 17 different cervical cancer cell lines; (B) PLK1 expression in cervical cancer and non-cancer tissues from pan-cancer projects.

A total of 17 cervical cancer cell lines including C-33 A, C-33-A, C-4-1, Ca Ski, Ca-Ski, DoTc2-4510, HeLa, HEp-2, HT-3, ME-180, MS751, OMC-1, SiHa, SISO, SKG-IIIa, SW756 and TC-YIK from the Cancer Genome project and 675 Genentech project were retrieved for PLK1 expression. PLK1 showed medium expression in all cervical cancer cell lines except TC-YIK cell line from Cancer Genome Project-cervical small cell carcinoma (Fig. 4A). As for pan-cancer analysis, PLK1 exhibited medium expression in cervical adenocarcinoma carcinoma and squamous cell carcinoma tissues, while PLK1 expression was below cutoff value in normal ectocervix and endocervix tissues (Fig. 4B).

### Verification from external microarrays and RNA-seq datasets

A total of 14 microarrays were included for expression analysis, including 367 cervical cancer samples and 151 non-cancerous cervical samples (Table 4). Among the 14 microarrays, expression matrix of PLK1 in CESC were extracted from seven microarrays including GSE138080, GSE27678-GPL570, GSE27678-GPL571, GSE52904- GPL6244, GSE63514, GSE7803 and GSE9750 as well as RNA-seq dataset from TCGA database. Expression matrix of PLK1 in cervical adenocarcinoma was extracted from GSE52904_GPL6244 and RNA-seq dataset from TCGA database. Expression pattern and discriminatory capacity of PLK1 in CESC, cervical adenocarcinoma and all types of cervical cancer was visualized in a panel of violin plots and ROC curves (Figs. 1–2, Fig. S1–S4). The pooled SMD and SROC curves indicated overexpression of PLK1 in CESC samples (SMD = 1.61, 95% CI [0.40–2.82]) and the significant power of PLK1 expression in distinguishing cervical cancer samples from non-cancer samples (AUC = 0.95) (Fig. S5), which was similar for PLK1 expression in all types of cervical cancer (Fig. S6).

### Integration of tissue microarray, external microarrays and RNA-seq datasets for comprehensive PLK1 expression analysis

**Figure 5:**
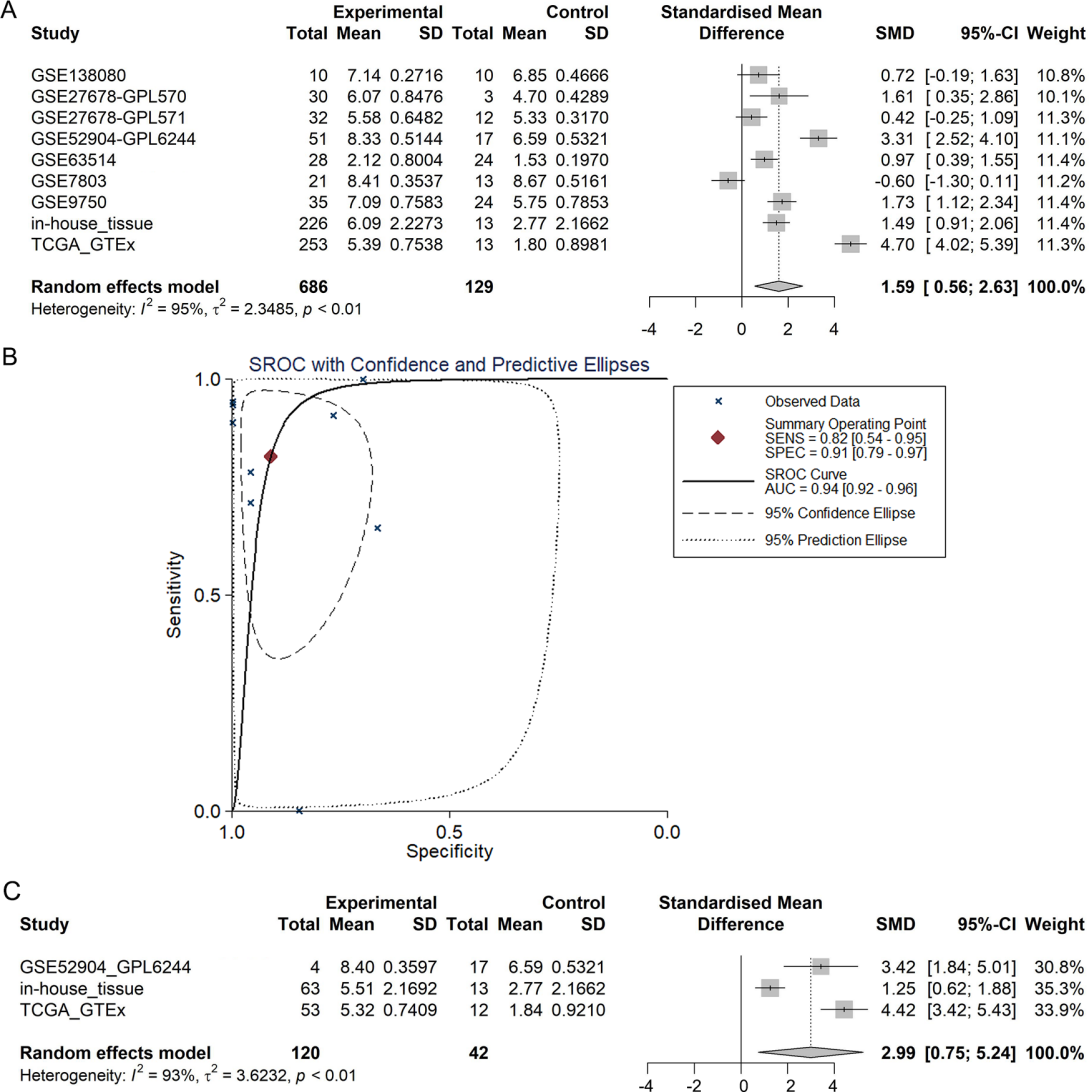
Pooled SMD forest plot and SROC curves of PLK1 in CESC and cervical adenocarcinoma for in-house tissue microarray, external microarrays and RNA-seq datasets. (A) SMD forest for CESC. (B) SROC curves for CESC. (C) SMD forest for cervical adenocarcinoma.

**Figure 6:**
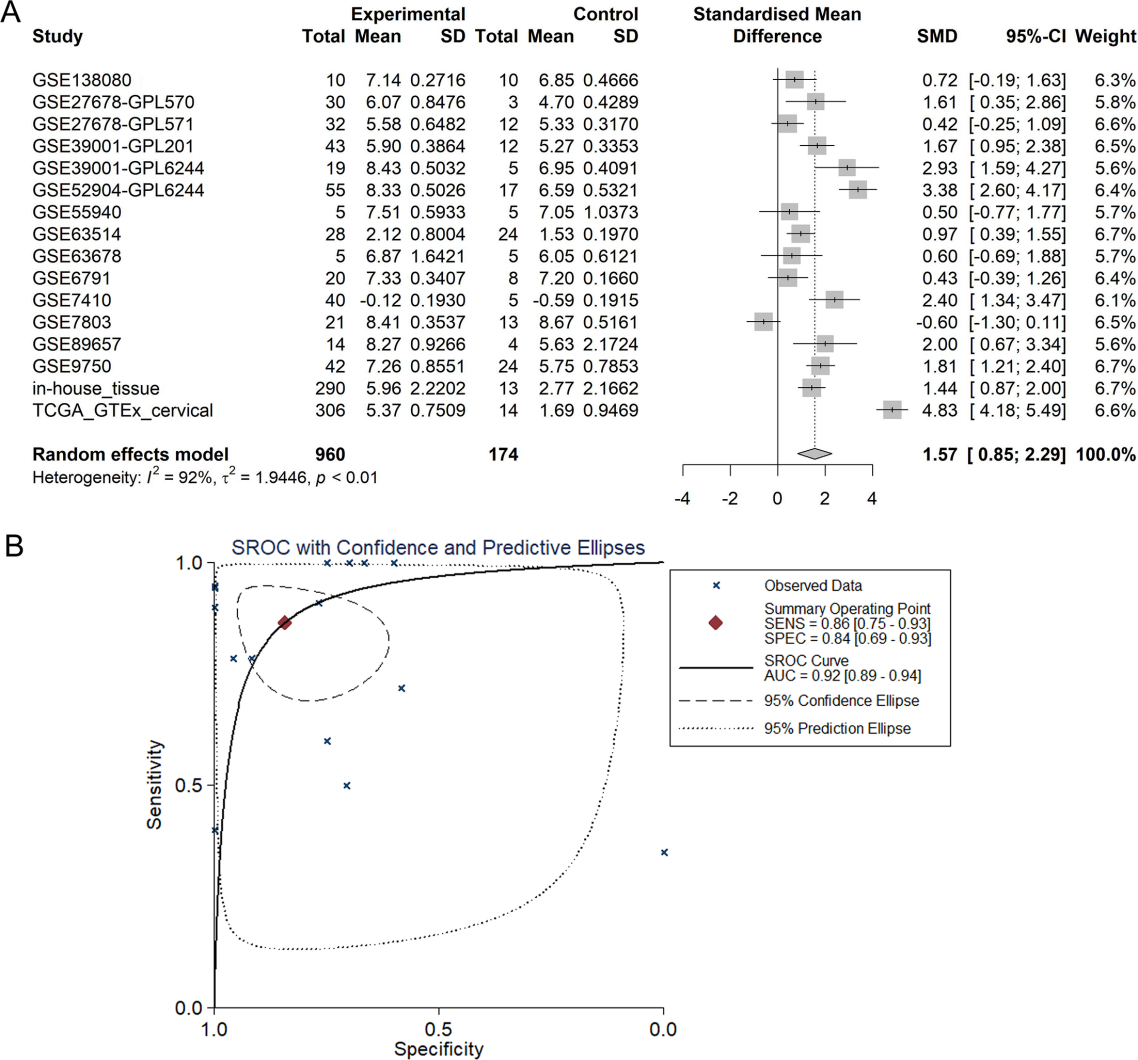
Pooled SMD forest plot and SROC curves of PLK1 in all types of cervical cancer for in-house tissue microarray, external microarrays and RNA-seq datasets. (A) SMD forest. (B) SROC curves.

The integrated SMD and SROC curves for CESC, cervical adenocarcinoma and all types of cervical cancer samples from in-house tissue microarray, external microarrays and RNA-seq datasets confirmed PLK1 overexpression in CESC, cervical adenocarcinoma and all types of cervical cancer (SMD = 1.59, 95% CI [0.56–2.63]; SMD = 2.99, 95% CI [0.75–5.24]; SMD = 1.57, 95% CI [0.85–2.29]) as well as the exceptional ability of PLK1 overexpression to discriminate CESC or all types of cervical cancer samples from non-cancer samples (AUC = 0.94; AUC = 0.92) (Figs. 5–6).

**Table 4:** Basic information for all included microarrays of PLK1.

ID	First author	Published year	Country	Platform	Cancer N	Cancer M	Cancer SD	Non-cancer N	Non-cancer M	Non-cancer SD	Sample type
GSE138080	Renske DM Steenbergen	2020	Netherlands	GPL23871	10	7.140	0.276	10	6.850	0.466	tissue
GSE27678	Ian Roberts	2013	United Kingdom	GPL570	30	6.060	0.853	3	4.700	0.474	tissue and cell
GSE27678	Ian Roberts	2013	United Kingdom	GPL571	32	5.580	0.650	12	5.330	0.316	tissue and cell
GSE39001	Ana María Espinosa	2013	Mexico	GPL201	43	5.900	0.386	12	5.260	0.334	tissue
GSE39001	Ana María Espinosa	2013	Mexico	GPL6244	19	8.430	0.507	5	6.950	0.412	tissue
GSE55940	chen ye	2014	China	GPL16238	5	7.510	0.594	5	7.050	1.039	tissue
GSE63514	Johan den Boon	2015	USA	GPL570	28	2.120	0.802	24	1.530	0.198	tissue
GSE63678	Prokopios Alexandros Polyzos	2015	USA	GPL571	5	6.870	1.645	5	6.050	0.622	tissue
GSE6791	Paul Ahlquist	2007	USA	GPL570	20	7.340	0.353	8	7.180	0.162	tissue
GSE7410	Petra Biewenga	2008	Netherlands	GPL1708	40	−0.110	0.200	5	−0.620	0.181	tissue
GSE7803	Rork Kuick	2007	USA	GPL96	24	8.520	0.496	17	8.370	0.353	tissue
GSE89657	Mauricio Salcedo Vargas	2016	Mexico	GPL6244	14	8.270	0.941	4	5.660	2.134	tissue
GSE9750	Murty Vundavalli	2008	USA	GPL96	42	7.260	0.858	24	5.750	0.784	tissue and cell
GSE52904	INGRID MEDINA MARTINEZ	2015	Mexico	GPL6244	55	8.339105527	0.521141083	17	6.604365294	0.526741331	tissue

**Notes.**

N number M mean SD standard deviation

### Prognostic value of PLK1 expression in cervical cancer

**Figure 7:**
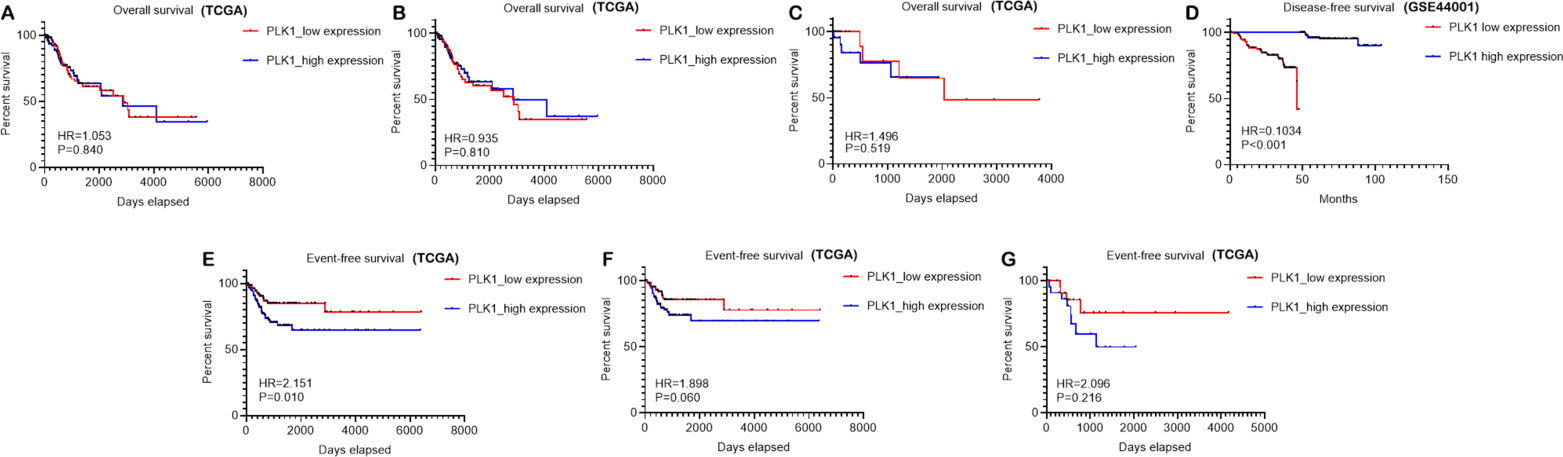
Survival analysis of PLK1 expression in cervical cancer from TCGA database and GSE44001. (A) Kaplan-Meier survival curves for overall survival of cervical cancer patients from TCGA database; (B) Kaplan-Meier survival curves for overall survival of CESC patients from TCGA database; (C) Kaplan-Meier survival curves for overall survival of cervical adenocarcinoma patients from TCGA database; (D) Kaplan-Meier survival curves for disease-free survival of all types of cervical cancer patients from GSE44001; (E) Kaplan-Meier survival curves for event-free survival of cervical cancer patients from TCGA database; (F) Kaplan-Meier survival curves for event-free survival of CESC patients from TCGA database; (G) Kaplan-Meier survival curves for event-free survival of cervical adenocarcinoma patients from TCGA database. TPM: transcripts per kilobase million; HR: hazard ratio

TCGA-CESC project and GSE44001 were included for survival analysis -because they contained survival information of cervical cancer patients. Kaplan–Meier survival curves for event-free survival of 285 all types of cervical cancer patients (233 CESC patients and 52 cervical adenocarcinoma patients) from TCGA database indicated that the event-free survival rate of cervical cancer patients with higher expression of PLK1 was shorter than that of patients with lower PLK1 (HR = 2.020, *P* = 0.0197) (Fig. 7C). An obvious trend was also shown in Fig. 7E and 7G that CESC patients or cervical adenocarcinoma patients from TCGA database with lower PLK1 expression experienced longer event-free survival time than those with higher PLK1 expression, though the results was not statistically significant.

### Mutation and alteration status of PLK1 in cervical cancer

**Figure 8:**
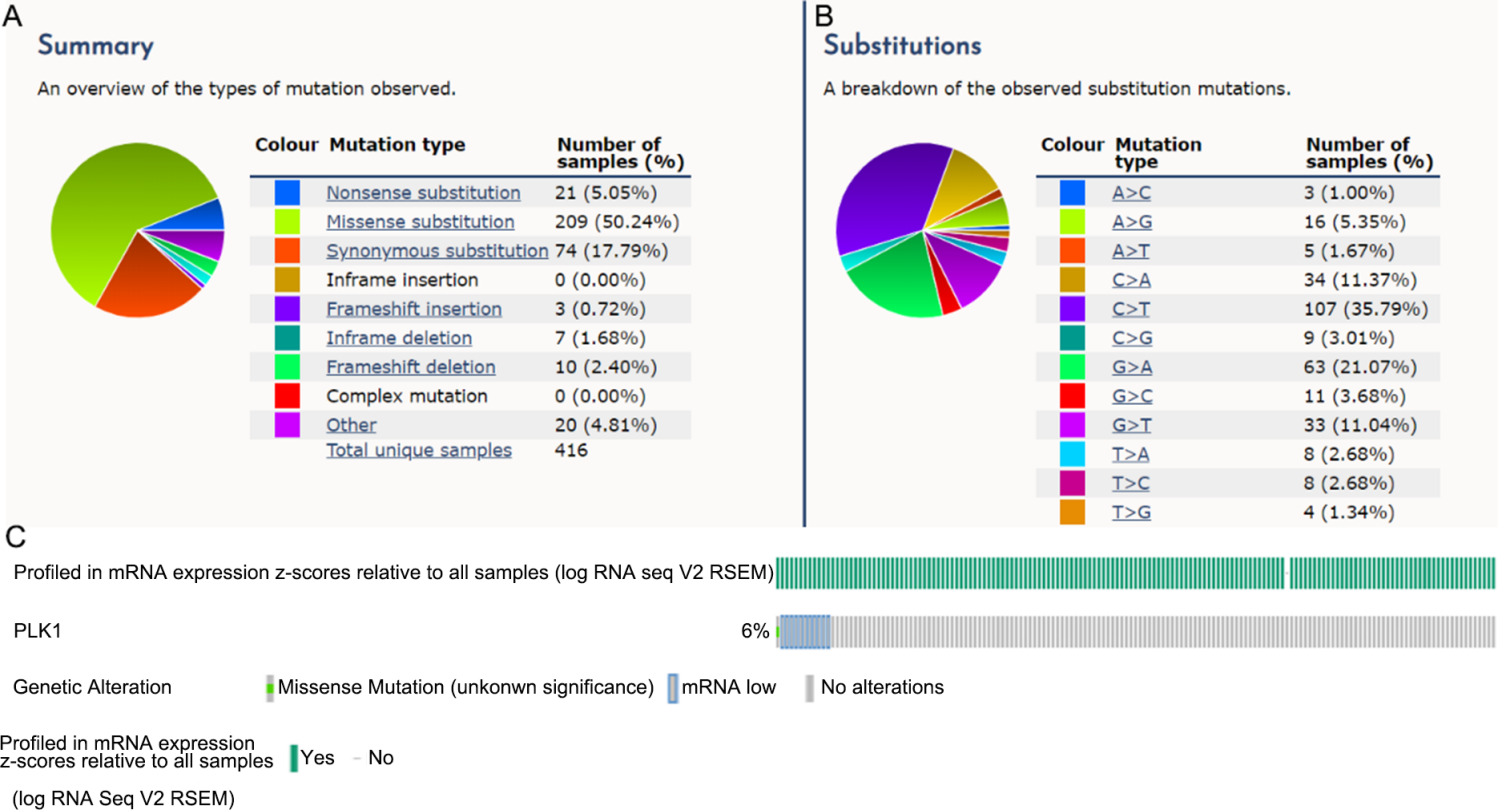
Mutation and genetic alteration of PLK1 in cervical cancer. (A) Pie chart of the mutation type of PLK1. (B) Breakdown of the substitution mutations. (C) Genetic alteration status of PLK1 in 190 cervical cancer samples profiled in mRNA expression.

According to Fig. 8, the predominant type of mutation for PLK1 was missense substitution, accounting for 50.24% of all samples, followed by synonymous substitution (Fig. 8A). Among all types of substitution mutations, the replacement of T by C was most commonly observed (35.79% of all samples) (Fig. 8B). A total of 12 cases (one case of missense mutation and 11 cases of mRNA low) with genetic alteration of PLK1 were recorded in 190 cervical cancer samples with mutation data from TCGA Firehose project (Fig. 8C). Summary of all cervical cancer and non-cancer samples included from different databases of the study was listed in Table 5.

**Table 5:** Summary of samples included from different databases.

Name	Link	This study	Included samples
TCGA	https://portal.gdc.cancer.gov/repository	To compare the expression of PLK1 in cervical cancer and non-cancer samples	306 cervical cancer and three non-cancer samples
GTEx	https://www.gtexportal.org/home/	To supplement the normal cervical samples of TCGA database	11 normal cervix samples
GEO	https://www.ncbi.nlm.nih.gov/gds/?term=	To compare the expression of PLK1 in cervical cancer and non-cancer samples	367 cervical cancer samples and 151 non-cancer cervical samples
Expression Atlas	https://www.ebi.ac.uk/gxa/home	To compare the expression of PLK1 in cervical cancer and non-cancer samples	17 cervical cancer cell lines and pan-cancer project of cervical cancer
cBioPortal	https://www.cbioportal.org/	To query genetic alteration profile of PLK1 in cervical cancer samples	190 cervical cancer samples

### Functional enrichment analysis for PLK1-correlated genes in cervical cancer

**Figure 9:**
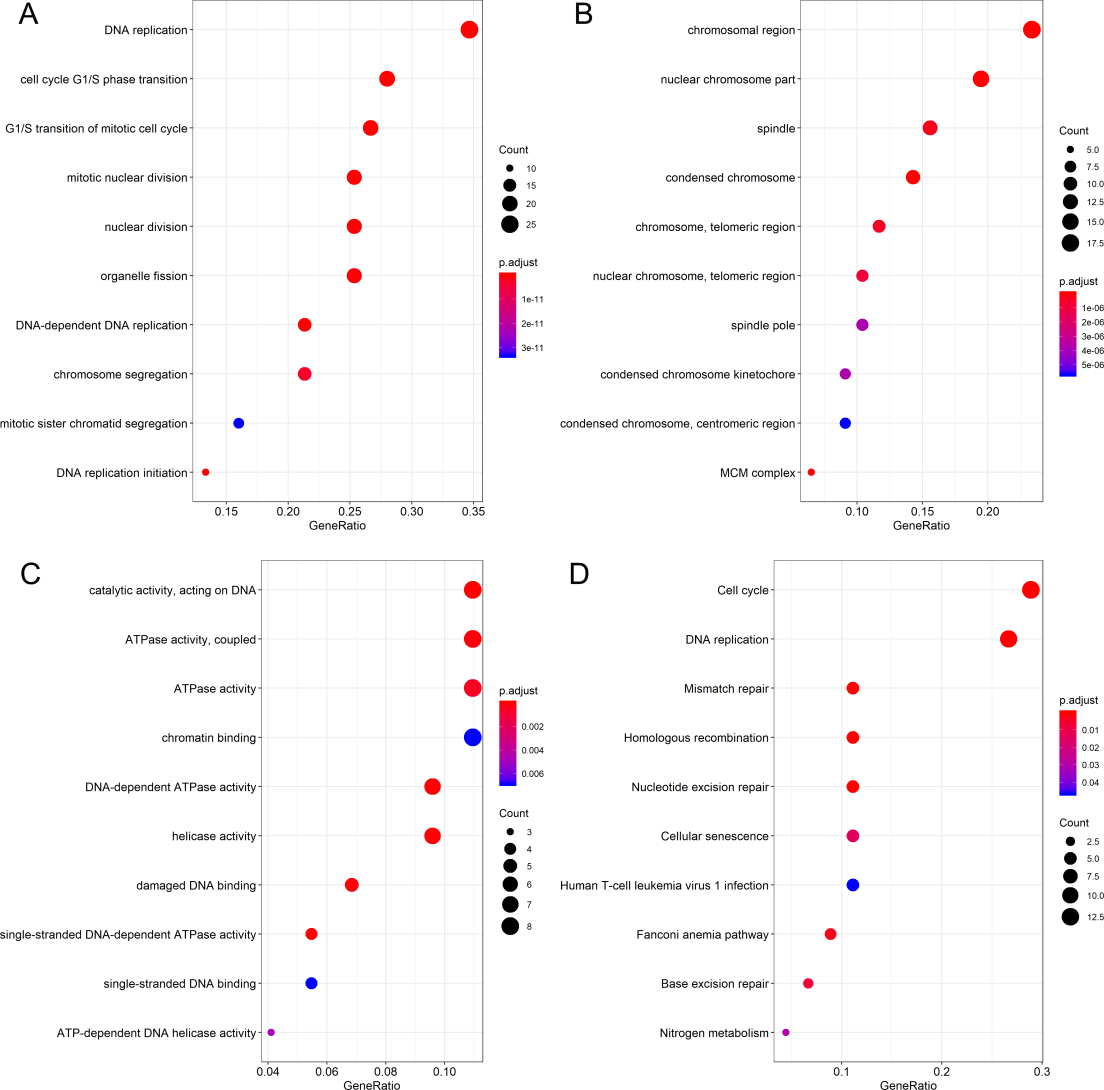
Functional enrichment analysis for genes positively correlated with PLK1 in CESC. (A) Dot plot for biological process terms. (B) Dot plot for cellular component terms. (C) Dot plot for molecular function terms. (D) Dot plot for pathway terms.

**Figure 10:**
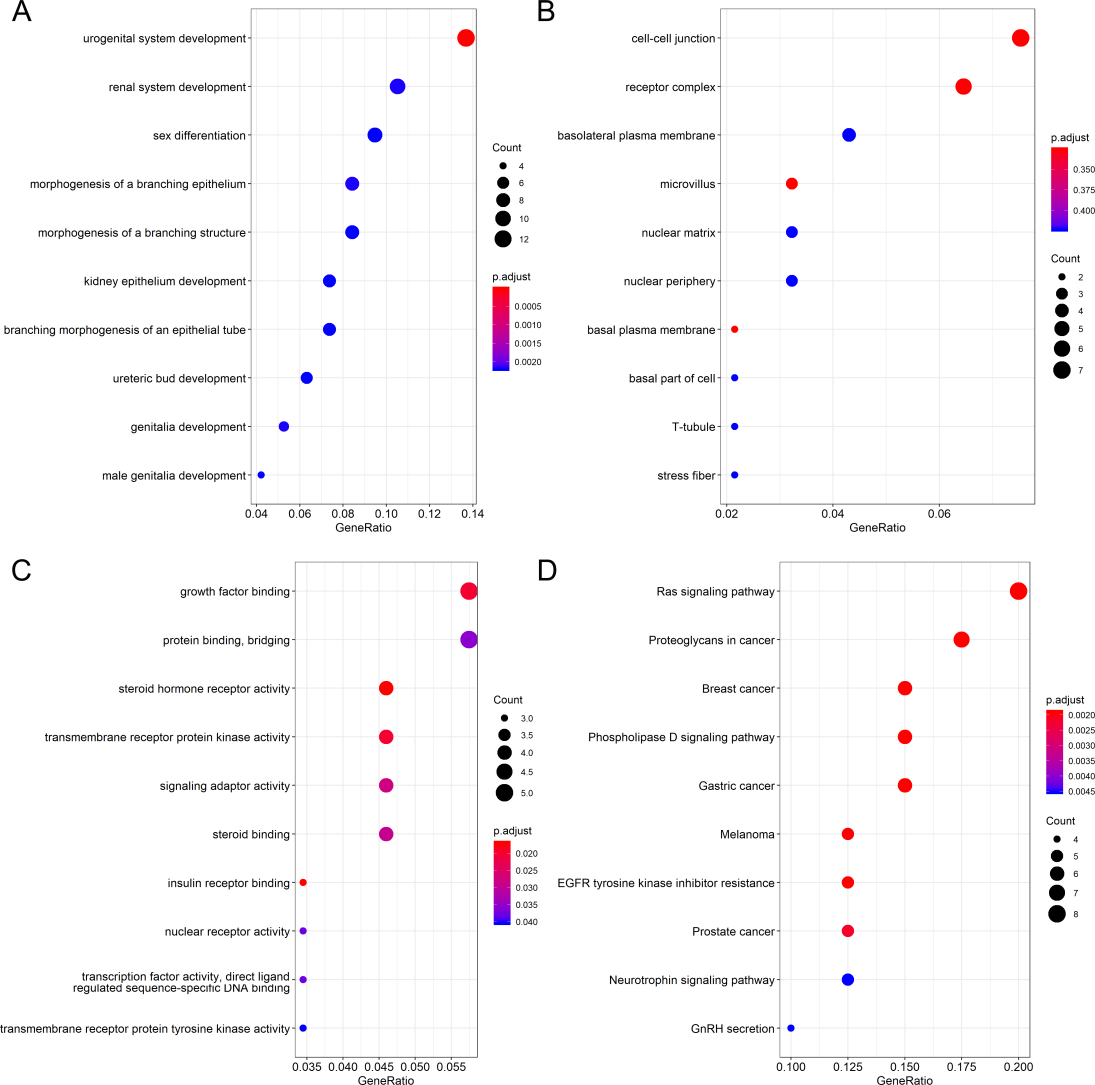
Functional enrichment analysis for genes negatively correlated with PLK1 in CESC. (A) Dot plot for biological process terms. (B)Dot plot for cellular component terms. (C) Dot plot for molecular function terms. (D) Dot plot for pathway terms.

**Figure 11:**
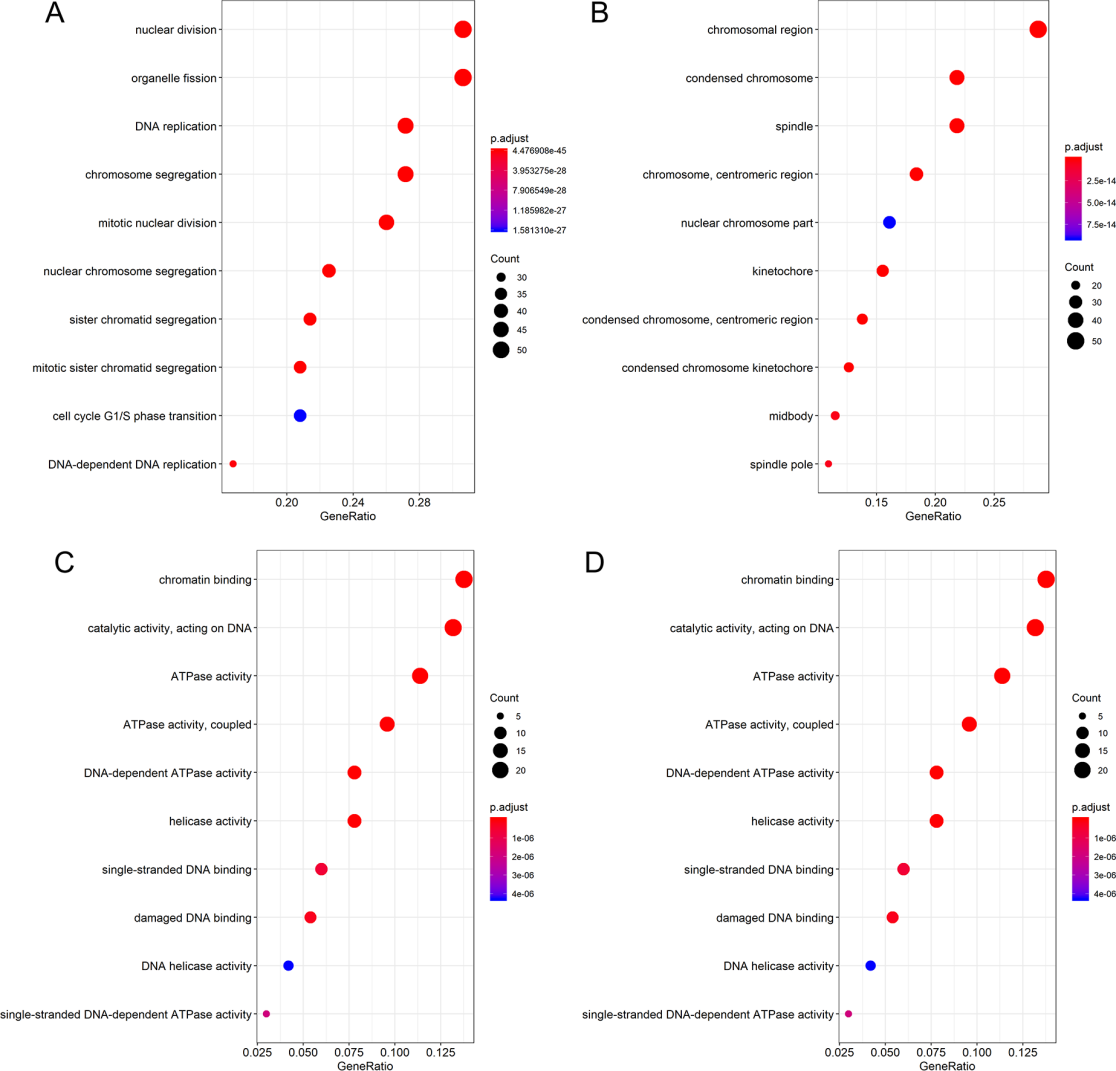
Functional enrichment analysis for genes positively correlated with PLK1 in cervical adenocarcinoma. (A) Dot plot for biological process terms. (B) Dot plot for cellular component terms. (C) Dot plot for molecular function terms. (D) Dot plot for pathway terms.

Differential expression analysis and Pearson correlation analysis were performed on seven microarrays of CESC (GSE138080, GSE27678-GPL570, GSE27678-GPL571, GSE52904-GPL6244, GSE63514, GSE7803 and GSE9750), one microarray of cervical adenocarcinoma (GSE52904- GPL6244) and RNA-seq dataset from TCGA database. Information of comparisons conducted in microarray data to get the DEG lists was listed in Table S1. The intersection of 455 upregulated DEGs and 371 genes that concurrently showed positive correlation with PLK1 (*r* > 0, adjusted *P* < 0.05) in at least three datasets generated 79 genes positively correlated with PLK1 in CESC; the intersection of 1,227 downregulated DEGs and 576 genes that concurrently showed negative correlation with PLK1 (*r* < 0, adjusted *P* < 0.05) in at least two datasets reported 100 genes negatively correlated with PLK1 in CESC (Figs. S7A and S7B). The intersection of 412 upregulated DEGs and 3898 genes that showed positive correlation with PLK1 (*r* > 0, adjusted *P* < 0.05) in RNA-seq dataset generated 177 genes positively correlated with PLK1 in cervical adenocarcinoma (Figs. S7C). No common part was found between 267 downregulated DEGs and 81 genes that showed negative correlation with PLK1 (*r* < 0, adjusted *P* < 0.05) in RNA-seq dataset of cervical adenocarcinoma. GO and KEGG analysis for 79 genes positively correlated with PLK1 in CESC demonstrated significant enrichment of these genes in biological processes including DNA replication, cell cycle G1/S phase transition and G1/S transition of mitotic cell cycle as well as pathways such as DNA replication, cell cycle, mismatch repair (Fig. 9) (Table S2). The 100 genes negatively correlated with PLK1 in CESC were significantly involved in biological processes including urogenital system development, renal system development and genitalia development as well as pathways such as Ras signaling pathway, melanoma and EGFR tyrosine kinase inhibitor resistance (Fig. 10) (Table S3). The 177 genes positively correlated with PLK1 in cervical adenocarcinoma were significantly involved in biological processes including nuclear division, DNA replication and organelle fission as well as pathways such as cell cycle, DNA replication and homologous recombination (Fig. 11) (Table S4).

## Discussion

In this study, tissue microarray, IHC and integrated microarrays as well as RNA-seq datasets were concomitantly used to investigate the clinic-pathological significance of PLK1 in cervical cancer including CESC and cervical adenocarcinomaas well as the genetic alteration profile of PLK1 and the functional enrichment of PLK1-related genes in cervical cancer, so as to provide insights on the clinical diagnosis and treatment of cervical cancer.

Although the expression and clinic-pathological value of PLK1 in cervical cancer have been studied, the research methods in previous studies were single and the number of samples is limited (Yang et al., 2017; Li et al., 2011; Zhang et al., 2009). The evaluation of clinic-pathological significance of PLK1 in cervical cancer by previous studies is incomplete, lacking assessment of prognostic value. In this study, a total of 963 cervical cancer samples and 178 non-cancer samples collected from in-house tissue microarrays and exterior microarrays and RNA-seq datasets were used for comprehensive assessment of the clinic-pathological significance of PLK1 in cervical cancer. We are the first group to compare PLK1 expression between CESC and cervical adenocarcinoma tissues and to evaluate PLK1 expression in CESC versus non-cancer and cervical adenocarcinoma versus non-cancer tissues. The results from progressive evidence chains constituted of tissue microarrays, IHC, exterior microarrays and RNA-seq dataset indicated PLK1 as a potential biomarker for cervical cancer.

It is worth noting that the high expression of PLK1 is significantly correlated with lymph node metastasis of CESC and all types of cervical cancer patients. Similar phenomena were found in other tumors. Previous researchers have demonstrated that overexpression of PLK1 is closely related to lymph node metastasis of gastric cancer, lung squamous cell carcinoma and breast cancer (Lin et al., 2017; Li et al., 2017; Zhang et al., 2017). Zhang et al. reported that STAT3 and PLK1 mutually control each other’s transcription in a positive feedback loop and activate the expression of each other to stimulate the growth of esophageal cancer cells (Zhang et al., 2012). The research work of Dang and other scholars proved that PLK1 may enhance the proliferation and migration of gastric cancer cells by participating in MEK-ERK pathway (Dang et al., 2018). Thus, it is speculated that PLK1 may also promote lymph node metastasis of cervical cancer by interacting with genes such as STAT3 or regulating certain pathways in cervical cancer.

With respect to the prognostic value of PLK1 in cervical cancer, we are the first group to appraise the expression of PLK1 on overall survival, disease-free survival, and event-free survival of CESC, cervical adenocarcinoma and all types of cervical cancer patients. The significantly prolonged event-free survival in patients with lower PLK1 expression implied potential prognostic stratification ability of PLK1 expression, which was consistent with the findings from the work by (Chhavi et al., 2010) that PLK1 expression was negatively correlated with the survival of malignant uterine cervix patients. We also noted that PLK1 expression exerted contradictory influence on survival of cervical cancer patients from GSE44001 and TCGA database, which might be explained by the difference of source of cervical cancer patients. Cervical cancer patients from GSE44001 were early cervical cancer patients who were treated with radical surgery with or without adjuvant therapies while TCGA-CESC project contained cervical cancer patients in all clinical stages and treatment status. The adverse influence of PLK1 expression on cervical cancer patients might be attributed to the promotive effect of PLK1 on the clinical progression of cervical cancer as discussed above.

In recent years, an increasing amount of evidence has revealed that miRNA participates in the occurrence and development of multiple human cancers by regulating target genes. The upstream regulation of miRNA might provide possible explanation for the molecular mechanism of PLK1 in cervical cancer. In cervical cancer, Li et al. found that miR-100 was down-regulated in cervical cancer and miR-100 negatively controlled the expression of PLK1 at the post-transcriptional level (Li et al., 2011). We conjectured that the overexpression of PLK1 in cervical cancer and the oncogenic influence of it on clinical progression of cervical cancer may result from the loss of miR-100 expression and negative transcriptional regulation.

We also endeavored to delve into the molecular mechanism of PLK1 in CESC and cervical adenocarcinoma through exploring the mutation and genetic alteration of PLK1 and functional enrichment analysis of correlated genes. Both mutation and genetic alteration profiles of PLK1 suggested that missense mutation occurred in PLK1 gene, which might explain the overexpression of PLK1 in cervical cancer. According to GO and KEGG analysis, biological processes, cellular component, molecular function, and pathway terms were differentially enriched between genes positively and negatively correlated with PLK1 in CESC and cervicla adenocarcinoma, which implied that genes positively and negatively correlated with PLK1 played various functions in development of CESC and cervical adenocarcinoma. Particularly, pathways such as cell cycle, DNA replication, homologous recombination were commonly shared by positively-correlated genes of PLK1 in CESC and cervical adenocarcinoma. It could be postulated that the three above pathways reflected the similarities of PLK1-centered molecular mechanism in CESC and cervical adenocarcinoma. The majority of the pathways significantly engaged by correlated genes such as cell cycle, DNA replication, homologous recombination, Ras signaling pathway and mismatch repair were closely associated with the initiation and progression of human cancers (Ingham & Schwartz, 2017; Kitao et al., 2018; Hoppe et al., 2018; Asati, Mahapatra & Bharti, 2016; Baretti & Le, 2018). It could be conjectured that PLK1-related carcinogenesis of CESC might relate to the active involvement of correlated genes in pathways such as DNA replication, cell cycle, mismatch repair and Ras signaling pathway; PLK1-related carcinogenesis of cervical adenocarcinoma might relate to the participation of correlated genes in pathways such as cell cycle, DNA replication, base excision repair and homologous recombination. Therapies targeting at these pathways might be beneficial to the treatment of cervical cancer patients.

Despite the inspiring discovery, limitations also existed in the current work. We emphasized on the clinic-pathological significance of PLK1 in cervical cancer in this study, in vitro and in vivo experiment was required in future work for further validating the functional role of PLK1 on biological process of cervical cancer. Although PLK1 expression in cervical cancer tissues was detected by multiple methods in the present study, the diagnostic value of PLK1 for cervical cancer embodied in differential expression level of circulating PLK1 between cervical cancer and non-cancer patients was not investigated, which was also not involved in literature studies pertaining PLK1 in other cancers. Collection of serum or plasma samples of cervical cancer and non-cancer patients was warranted for appraising diagnostic value of PLK1 for cervical cancer. Furthermore, nanoparticles targeting PLK1 in serum of cervical cancer tissues possess unlimited potential of clinical diagnose and treatment for cervical cancer. Great efforts will be committed to developing nanovectors directed against PLK1 for improved diagnose and treatment of cervical cancer.

## Conclusions

To sum up, we proved overexpression of PLK1 in CESC versus non-cancer and cervical adenocarcinoma versus non-cancer samples via multiple detection technologies. The high expression of PLK1 is significantly correlated with the clinical progression of CESC. PLK1-related carcinogenesis of cervical cancer might relate to the active involvement of correlated genes in pathways such as DNA replication, cell cycle, mismatch repair and homologous recombination. The overexpression of PLK1 in cervical cancer and the contributory effect of it on clinical progression indicated the hopeful prospect of PLK1 as a biomarker for cervical cancer.

##  Supplemental Information

10.7717/peerj.10458/supp-1 Supplemental Information 1 PLK1 expression in CESC and non-cancer samples from in-house tissue microarrays, external microarrays and RNA-seq dataset

10.7717/peerj.10458/supp-2 Supplemental Information 2 PLK1 expression in cervical adenocarcinoma and non-cancer samples from in-house tissue microarrays, external microarrays and RNA-seq dataset

10.7717/peerj.10458/supp-3 Supplemental Information 3 Gene ontology and Kyoto Encyclopedia of Genes and Genomes pathway analysis for genes negatively correlated with PLK1 in CESC

10.7717/peerj.10458/supp-4 Supplemental Information 4 The discriminatory ability of PLK1 expression in distinguishing cervical adenocarcinoma from non-cancer tissues in each microarray and RNA-seq dataset

10.7717/peerj.10458/supp-5 Supplemental Information 5 Pooled SMD forest plot and SROC curves of PLK1 in CESC for all included microarrays and RNA-seq datasets

10.7717/peerj.10458/supp-6 Supplemental Information 6 Pooled SMD forest plot and SROC curves of PLK1 in all types of cervical cancer for all included microarrays and RNA-seq datasets

10.7717/peerj.10458/supp-7 Supplemental Information 7 Venn plots of genes positively or negatively correlated with PLK1

10.7717/peerj.10458/supp-8 Supplemental Information 8 Information of comparisons conducted in microarray data to get the DEG lists

10.7717/peerj.10458/supp-9 Supplemental Information 9 Gene ontology and Kyoto Encyclopedia of Genes and Genomes pathway analysis for genes positively correlated with PLK1 in CESC

10.7717/peerj.10458/supp-10 Supplemental Information 10 Gene ontology and Kyoto Encyclopedia of Genes and Genomes pathway analysis for genes positively correlated with PLK1 in cervical adenocarcionma

10.7717/peerj.10458/supp-11 Supplemental Information 11 Gene ontology and Kyoto Encyclopedia of Genes and Genomes pathway analysis for genes positively correlated with PLK1 in cervical adenocarcionma

## Additional Information and Declarations

### Data Availability

The following information was supplied regarding data availability: The data is available at FigShare: Gao, Li (2020): Raw code in R. figshare. Dataset. https://doi.org/10.6084/m9.figshare.12751448.v1
Gao, Li (2020): Raw data of survival analysis. figshare. Dataset. https://doi.org/10.6084/m9.figshare.12751439.v2
Gao, Li (2020): Raw data of in-house tissue microarray. figshare. Dataset. https://doi.org/10.6084/m9.figshare.12751433.v2
Gao, Li (2020): Clinical information of cervical cancer patients from TCGA. figshare. Dataset. https://doi.org/10.6084/m9.figshare.12751430.v1
Gao, Li (2020): Gene matrix for cervical adenocarcinoma. figshare. Dataset. https://doi.org/10.6084/m9.figshare.12751427.v1
Gao, Li (2020): Gene matrix for CESC. figshare. Dataset. https://doi.org/10.6084/m9.figshare.12751418.v1
Gao, Li (2020): Gene matrix for all types of cervical cancer.txt. figshare. Dataset. https://doi.org/10.6084/m9.figshare.12751415.v1.
