# Supplementary figures and images for: Polo like kinase 1 expression in cervical cancer tissues generated from multiple detection methods
Source: PeerJ. 2020 Dec 8;8:e10458. doi: 10.7717/peerj.10458 (PMC7731657; doi:10.7717/peerj.10458)

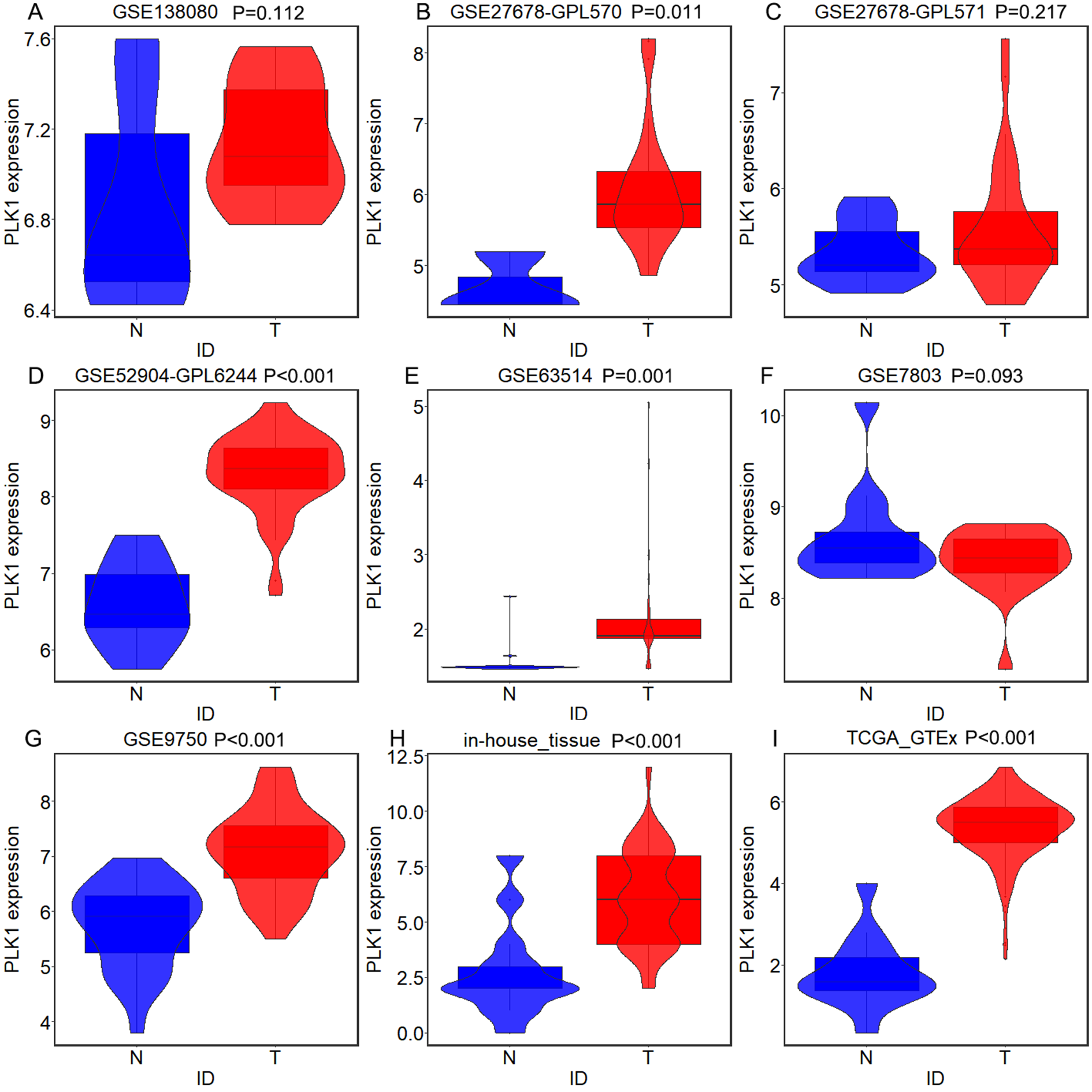

Supplement: Supplemental Information 1 [file peerj-08-10458-s001.png]

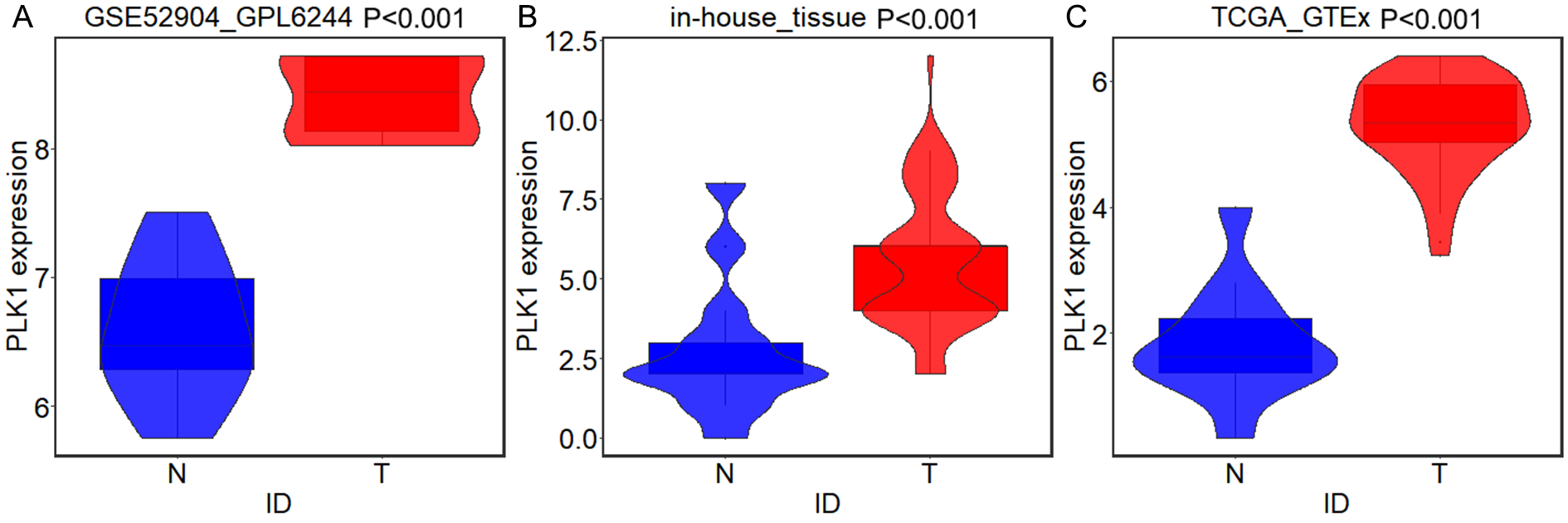

Supplement: Supplemental Information 2 [file peerj-08-10458-s002.png]

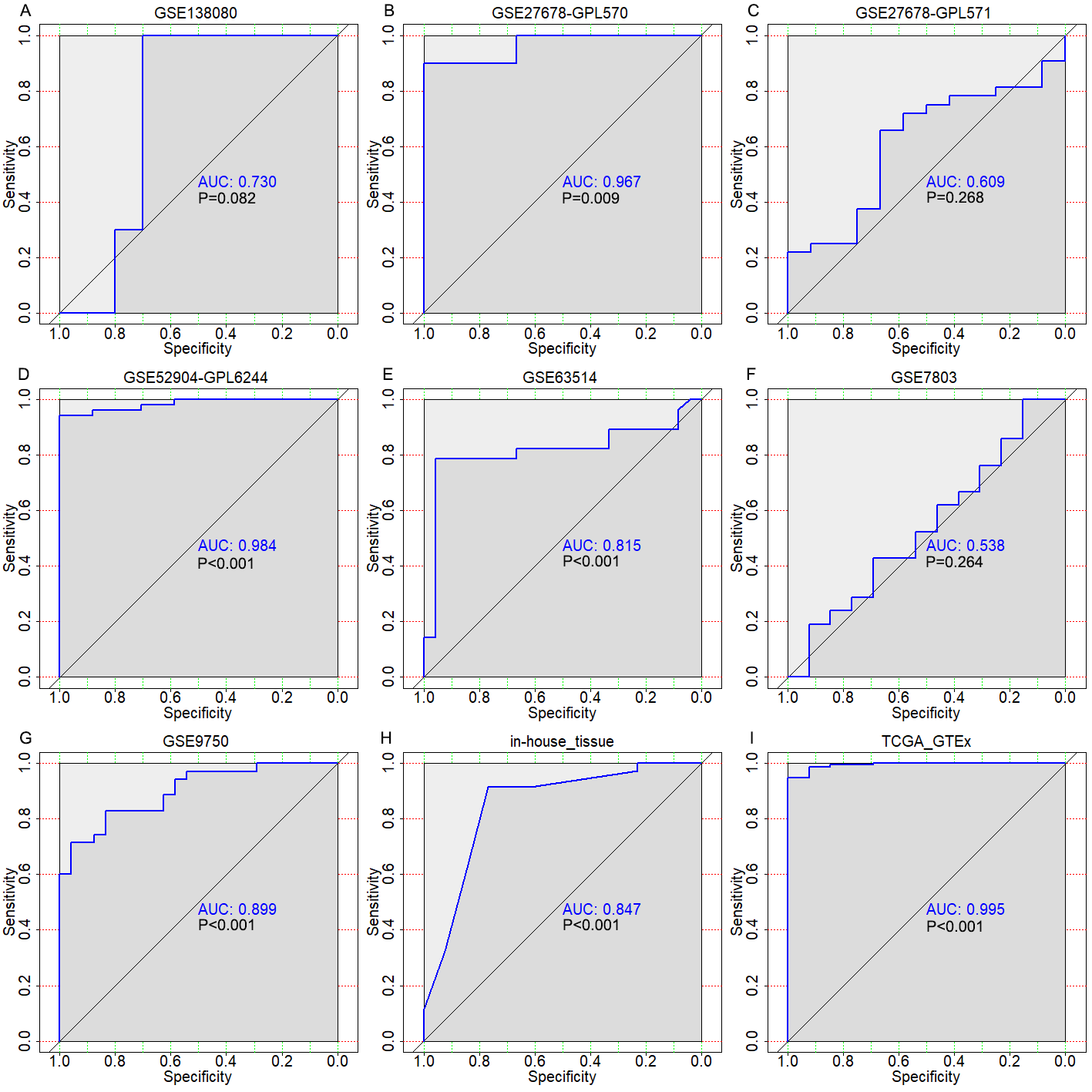

Supplement: Supplemental Information 3 [file peerj-08-10458-s003.png]

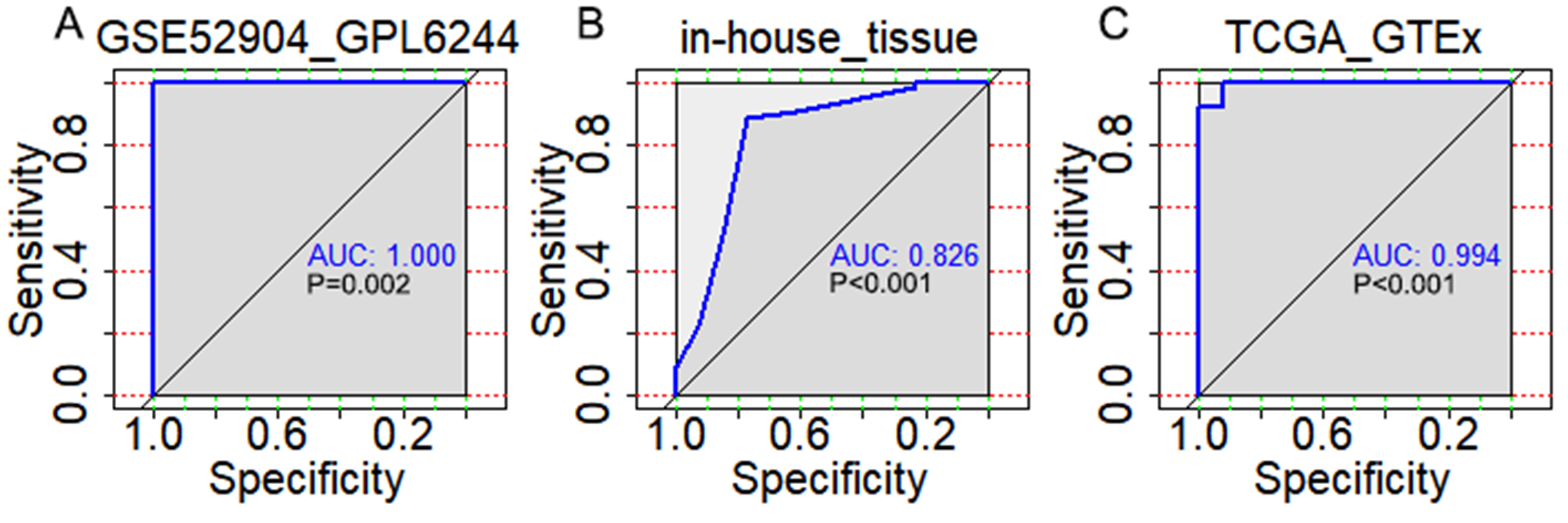

Supplement: Supplemental Information 4 [file peerj-08-10458-s004.png]

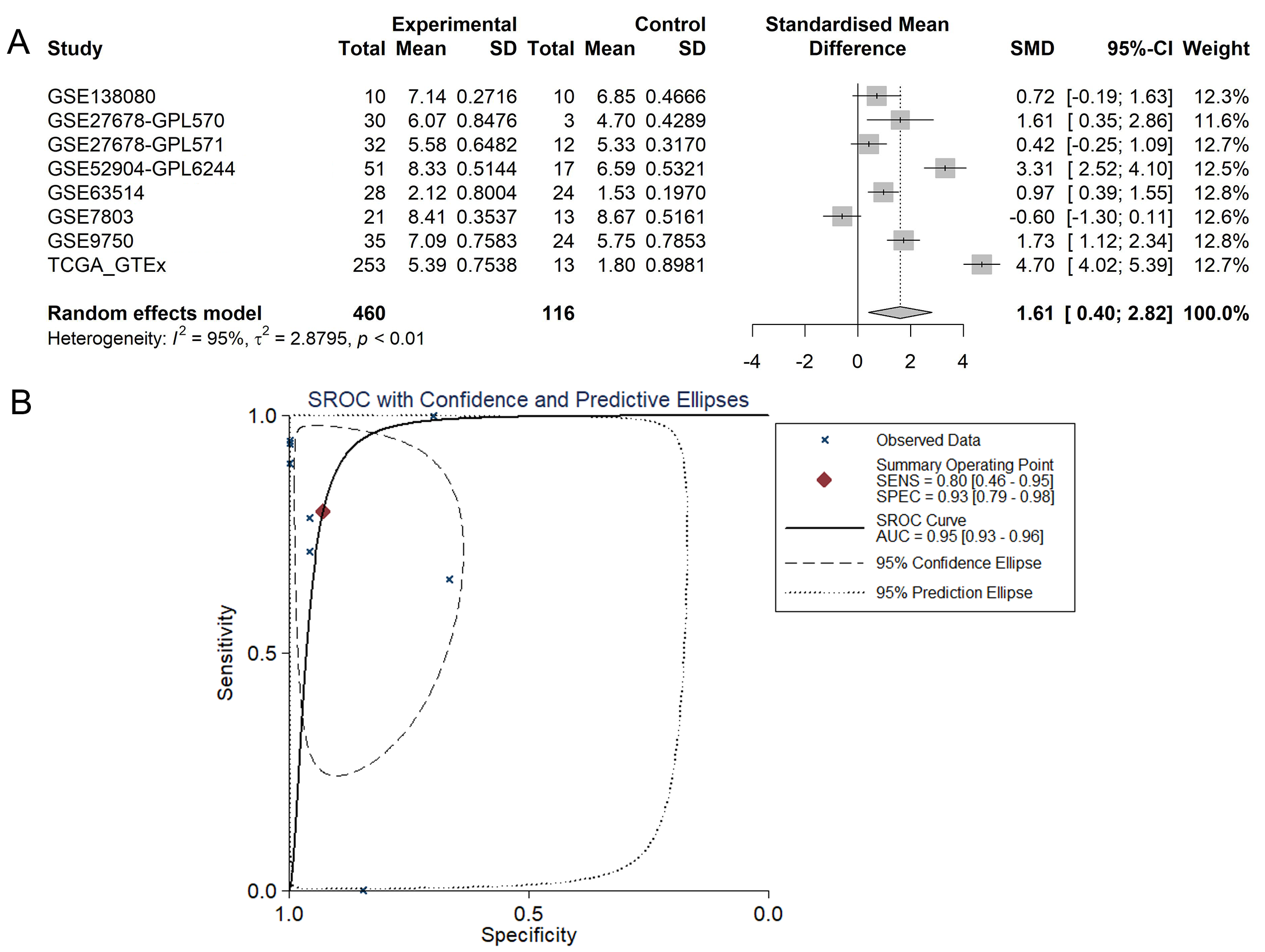

Supplement: Supplemental Information 5 [file peerj-08-10458-s005.png]

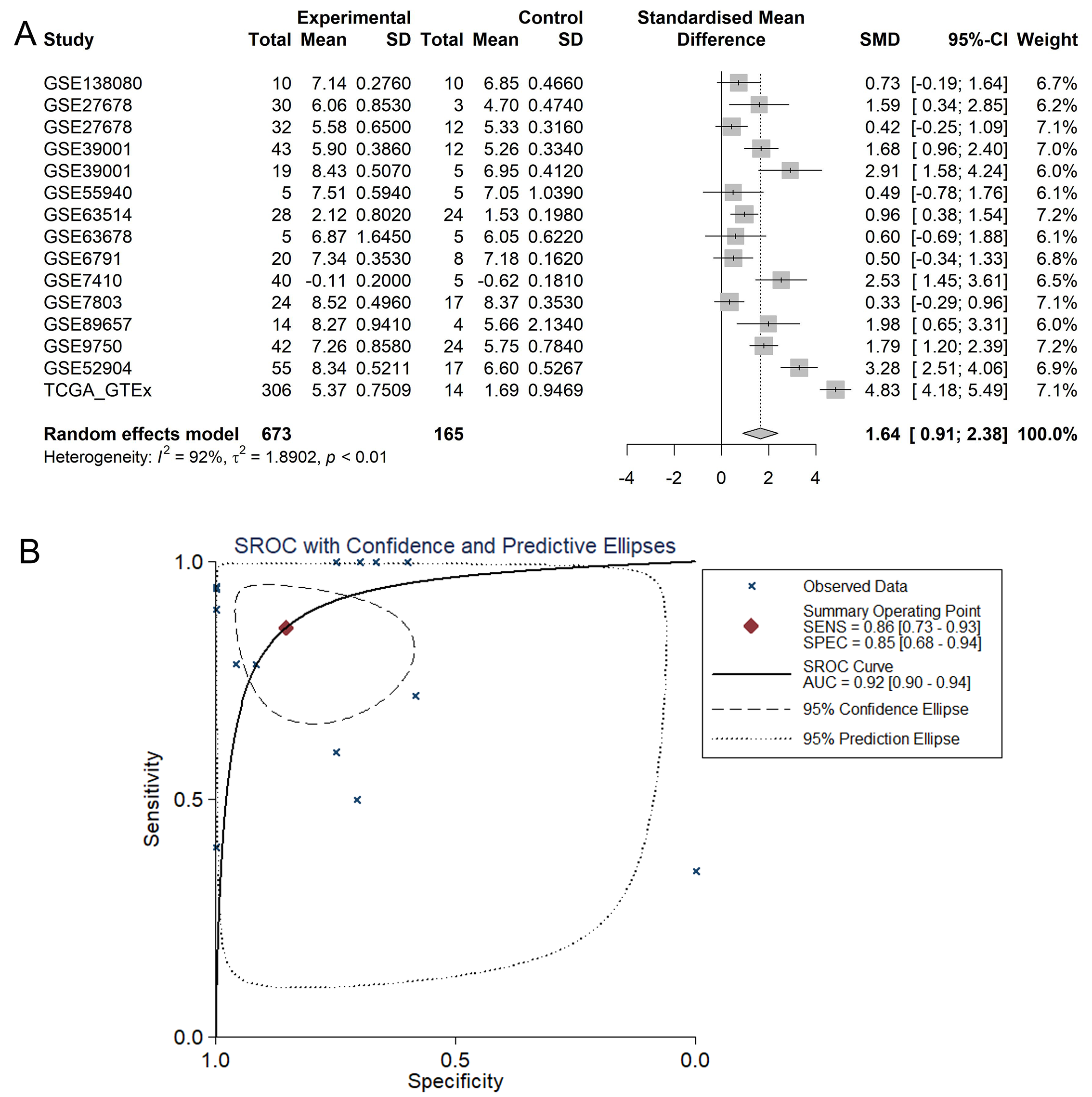

Supplement: Supplemental Information 6 [file peerj-08-10458-s006.png]

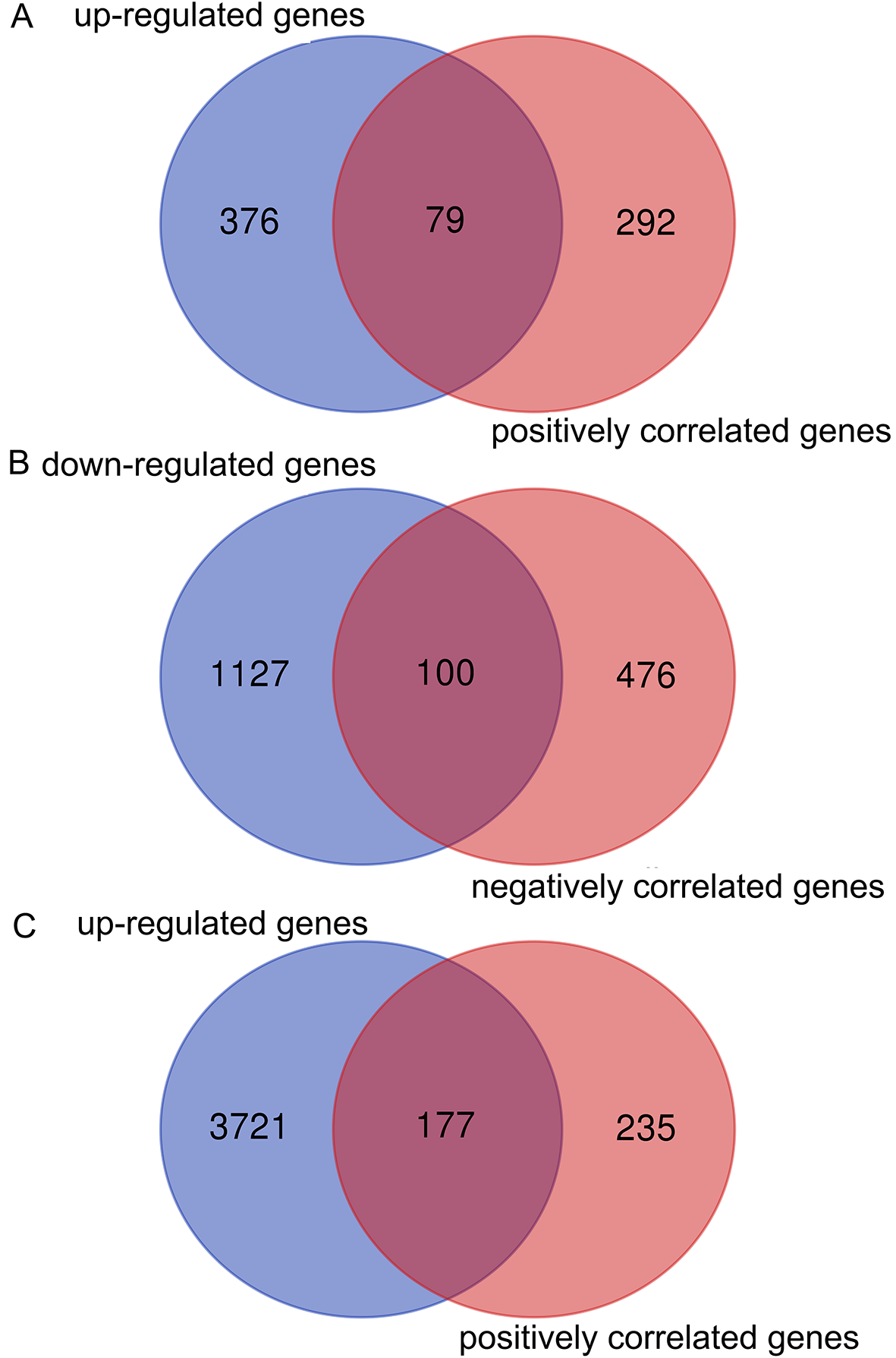

Supplement: Supplemental Information 7 [file peerj-08-10458-s007.png]
